# Pharmacological AhR activation attenuates airway hyperresponsiveness and remodeling in a mixed allergen asthma model

**DOI:** 10.1016/j.isci.2026.117842

**Published:** 2026-10-17

**Authors:** Mohammad Irshad Reza, Premanand Balraj, Ashish Kumar, Kathryn M. Heyob, Joshua Walum, Rodney D. Britt, Venkatachalem Sathish

**Affiliations:** 1 Division of Pulmonary, Critical Care & Sleep Medicine, Department of Internal Medicine, The University of Oklahoma Health Campus, Oklahoma City, OK, USA; 2 Department of Pharmaceutical Sciences, North Dakota State University, Fargo, ND, USA; 3 Center for Perinatal Research, Abigail Wexner Research Institute at Nationwide Children’s Hospital, Columbus, OH, USA; 4 Department of Pediatrics, The Ohio State University, Columbus, OH, USA

**Keywords:** asthma, airway hyperresponsiveness, airway remodeling, aryl hydrocarbon receptor, AhR, mixed allergen, airway smooth muscle, RNA seq

## Abstract

Airway hyperresponsiveness and remodeling are major features of asthma. We investigated whether pharmacological activation of the aryl hydrocarbon receptor (AhR) modifies these responses in a mixed allergen (MA) mouse model of asthma. MA exposure increased airway resistance, contractility, inflammation, and remodeling, with elevated AhR expression in whole lung and airway smooth muscle but limited canonical AhR target gene activation. Treatment with the high-affinity AhR agonist 2,3,7,8-tetrachlorodibenzo-p-dioxin (TCDD) improved lung mechanics, reduced inflammatory cell and cytokine accumulation, attenuated airway remodeling, and decreased airway narrowing in precision-cut lung slices. Bulk lung RNA sequencing further showed suppression of inflammatory, neutrophil-associated, and remodeling pathways following TCDD treatment, together with induction of canonical AhR target genes. Collectively, these findings demonstrate that AhR activation suppresses inflammatory gene networks, limits airway remodeling, and improves airway mechanics in experimental asthma, supporting the therapeutic potential of targeting AhR signaling to mitigate airway dysfunction and structural remodeling.

## Highlights

• Chronic allergen exposure upregulates AhR in airway smooth muscle
• AhR activation attenuates airway remodeling in chronic asthma
• AhR activation reduces airway hyperresponsiveness and contractility *in vivo*
• AhR suppresses pro-inflammatory and ECM remodeling pathways

## Graphical abstract

**Figure:**
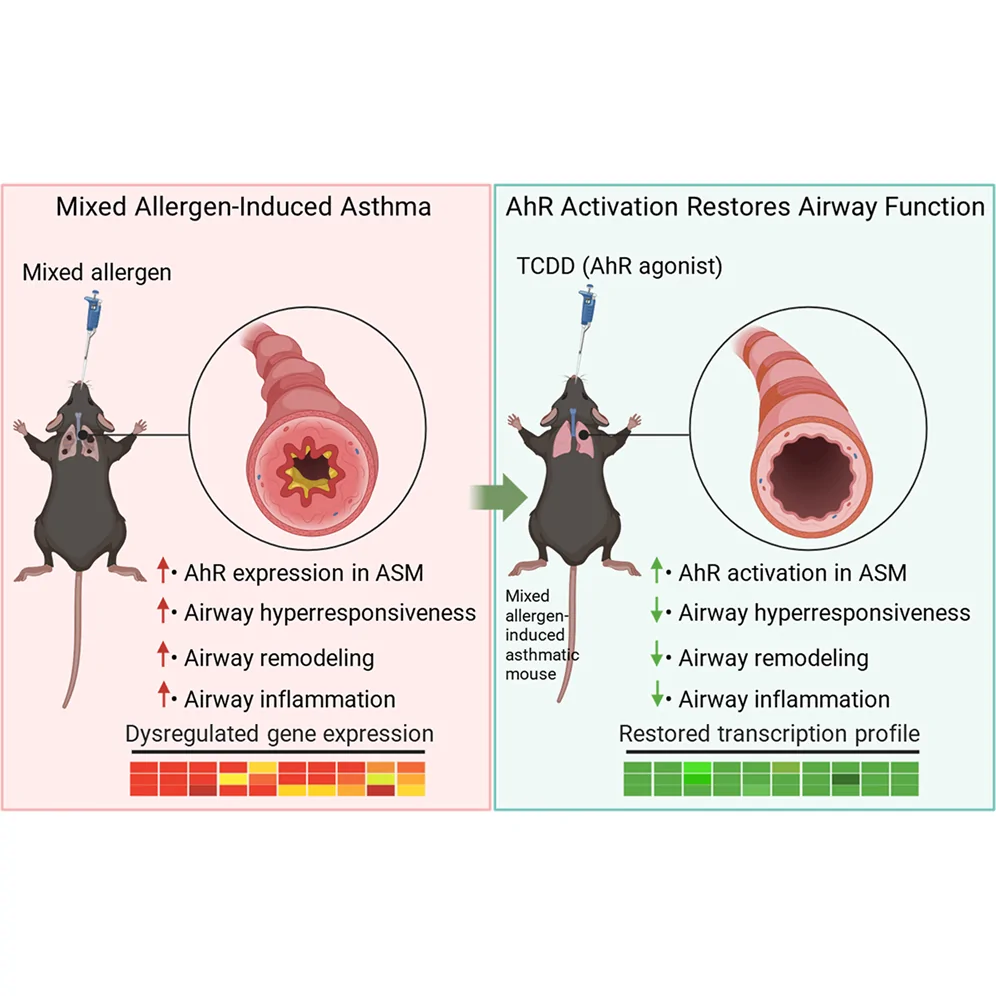

## Teaser

Respiratory medicine; Therapeutics; Immune response

## Introduction

Asthma is one of the most prevalent chronic inflammatory diseases globally.^1^^,^^2^^,^^3^ In the United States alone, approximately 25 million individuals are affected, with an estimated mortality rate of 10.6 deaths per million population.^4^^,^^5^ Airway remodeling is a defining feature of asthma and encompasses a range of structural changes within the airway wall, including subepithelial fibrosis, expansion of airway smooth muscle (ASM), goblet cell hyperplasia, angiogenesis, and excessive deposition of extracellular matrix (ECM) components.^1^^,^^6^^,^^7^^,^^8^^,^^9^ These changes are driven by a complex interplay of chronic inflammation, proliferation and migration of structural cells, ECM accumulation, and enhanced airway contractility.^10^^,^^11^^,^^12^^,^^13^ Collectively, these processes lead to airway wall thickening, mucus hypersecretion, airway hyperresponsiveness, and a progressive decline in lung function.^8^^,^^14^^,^^15^ Despite advances in asthma management, current therapeutic strategies fail to adequately prevent or reverse airway remodeling, resulting in poor disease control. This underscores the urgent need for deeper mechanistic understanding of airway remodeling and the identification of novel therapeutic targets.

The aryl hydrocarbon receptor (AhR) is a ligand-activated transcription factor traditionally recognized for mediating cellular responses to environmental toxins.^16^^,^^17^ Acting as a sensor for environmental pollutants, dietary metabolites, and endogenous ligands, AhR regulates diverse cellular and physiological processes through complex, ligand- and cell-specific transcriptional programs.^18^^,^^19^ Beyond its classical role in xenobiotic metabolism, emerging evidence implicate AhR in immune regulation, inflammation, and maintenance of cellular homeostasis.^20^^,^^21^^,^^22^ In the respiratory system, AhR plays a pivotal role in preserving epithelial barrier integrity,^21^^,^^23^ regulating smooth muscle cell phenotypes,^24^^,^^25^^,^^26^^,^^27^ and modulating inflammatory responses, highlighting its potential as a therapeutic target in asthma and airway remodeling.^28^ Previous studies have demonstrated that genetic deletion of *Ahr* exacerbates inflammatory responses in pulmonary diseases,^29^ suggesting its anti-inflammatory role in airway inflammation. Conversely, AhR activation has been shown to mitigate hypoxia-induced airway inflammation, restore immune balance, and improve lung function.^30^

Our recent work identified AhR expression in the human lung, particularly within the ASM layer,^27^ suggesting a functional role of AhR signaling in the airways. We further observed that AhR expression is elevated in ASM cells derived from asthmatic patients and during inflammatory conditions.^27^ Importantly, activation of AhR by its endogenous ligand inhibited inflammation-induced mitochondrial fission in ASM cells through transcriptional suppression of *DRP1*.^31^^,^^32^ However, the *in vivo* relevance of AhR signaling in asthma pathophysiology, particularly in the context of airway remodeling and airway hyperresponsiveness, remains unexplored. Moreover, the molecular signatures regulated by AhR activation in allergen-driven asthma have not been systematically defined at the transcriptomic level. To bridge these gaps and extend our *in vitro* findings, we employed a mixed allergen (MA)-induced mouse model of chronic asthma to investigate the impact of AhR activation on airway physiology. Our findings provide the first *in vivo* evidence that pharmacological AhR activation with 2,3,7,8-tetrachlorodibenzo-p-dioxin (TCDD) confers protective effects in ASM against chronic MA-induced airway dysfunction, thereby establishing a mechanistic link between AhR signaling and airway remodeling in asthma. Furthermore, we provide differential gene expression analysis to reveal the AhR-mitigated transcriptional changes and the inflammation/remodeling pathways in the lung during asthma.

## Results

### AhR activation improves lung function in MA-induced mouse model of asthma

The experimental design of the study is illustrated in Figure 1A. Initially, we evaluated the effect TCDD, an established AhR ligand, on the impaired lung function in the MA-induced mouse model of asthma. Lung function parameters, including total respiratory system resistance (Rrs), respiratory system elastance (Ers), and respiratory system compliance (Crs), were measured using the forced oscillation technique on the flexiVent (SCIREQ). MA-exposed mice exhibited increased Rrs and Ers, along with reduced Crs, in response to increasing concentrations of methacholine (MCh, 0–50 mg/mL) compared with phosphate-buffered saline (PBS)-challenged controls (Figures 1B–1D). To determine treatment efficacy, we analyzed the maximal response (Max Rrs, Ers, and Crs) at the highest MCh concentration (50 mg/mL, Figures 1E–1G) and area under curve (AUC, Figures 1H–1J). MA-challenged mice demonstrated a significant increase in Rrs and Ers and a decline in Crs. Notably, mice treated with TCDD alone did not show significant changes in any of these parameters. However, MA-challenged mice treated with TCDD displayed a significant reduction in Rrs and Ers and an improvement in Crs compared with MA-challenged mice (Figures 1B–1J). As both male and female mice were included in the study, an exploratory sex-stratified analysis was performed for the primary lung function outcomes. Two-way ANOVA revealed no significant treatment and sex interaction for Rrs or Crs. Consistent with the overall findings, TCDD treatment reduced airway resistance and improved lung compliance in both male and female mice (Figure S3).

**Figure 1:**
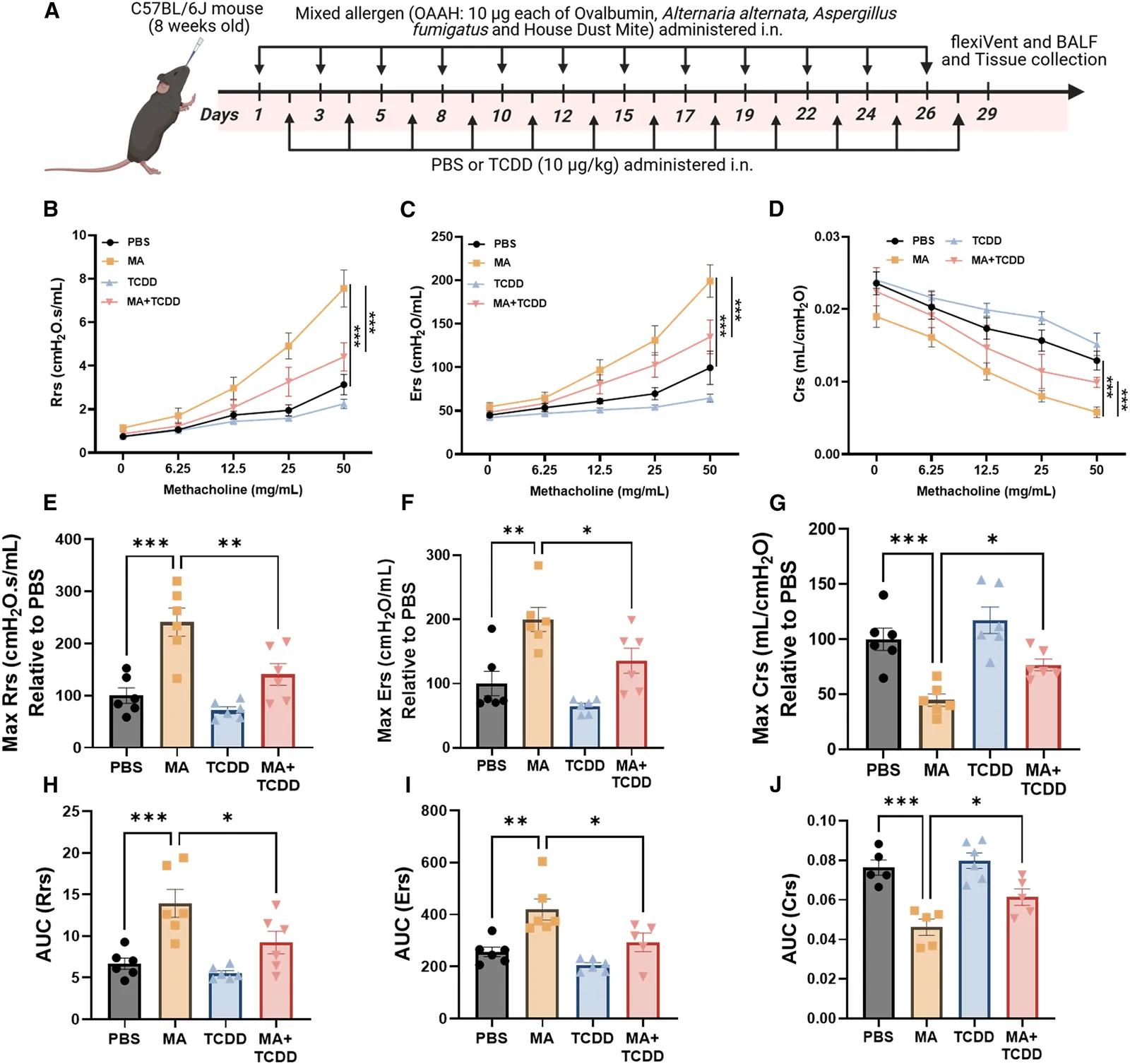
Effect of AhR activation on the impaired lung function in the MA-induced mouse model of asthma (A) Schematic representation of the experimental design showing MA sensitization followed by TCDD treatment and lung function assessment. (B–D) Lung function parameters including total respiratory system resistance (Rrs), elastance (Ers), and compliance were measured in response to increasing concentrations of methacholine (MCh, 0–50 mg/mL) using the flexiVent. (E–G) Maximal Rrs (Max Rrs), Ers (Max Ers), Crs (Max Crs) at 50 mg/mL MCh and (H–J) Area under curve (AUC) Rrs, Ers, Crs were used to compare treatment efficacy. MA-challenged mice showed a significant increase in Rrs and Ers with a concomitant decrease in Crs compared with PBS controls. TCDD treatment alone did not alter lung function; however, TCDD administration in MA-challenged mice significantly reduced Rrs and Ers and improved Crs compared with MA-challenged mice. Data were evaluated using repeated measures one/two-way ANOVA with Tukey’s post hoc test for multiple comparisons. Mean ± SEM (*n* = 5–6 mice/group); ∗*p* < 0.05, ∗∗*p* < 0.01, ∗∗∗*p* < 0.001.

### AhR activation reduces leukocytes accumulation and inflammatory cytokine levels in the BALF of MA-induced asthmatic mice

To evaluate the effect of AhR activation on the inflammatory milieu, immune cells, and cytokine levels were quantified in the bronchoalveolar lavage fluid (BALF). MA-challenged mice exhibited a significant increase in total bronchoalveolar lavage (BAL) cells (Figure 2A), leukocytes: neutrophils, eosinophils, and macrophages (Figure 2B), and multiple cytokines, including IL-1β (Figure 2C), IL-5 (Figure 2D), IL-6 (Figure 2E), TNF-α (Figure 2F), IL-17 (Figure 2G), monocyte chemoattractant protein-1 (MCP-1) (Figure 2H), eotaxin (Figure 2I), granulocyte colony-stimulating factor (G-CSF) (Figure 2J), keratinocyte-derived chemokine (KC) (Figure 2K), and leukemia inhibitory factor (LIF) (Figure 2L), compared with PBS controls. Treatment with TCDD alone did not significantly affect cytokine levels compared to the PBS group. However, TCDD administration in MA-challenged mice significantly suppressed the MA-induced increase in all cytokines level, indicating a strong anti-inflammatory effect mediated by AhR activation.

**Figure 2:**
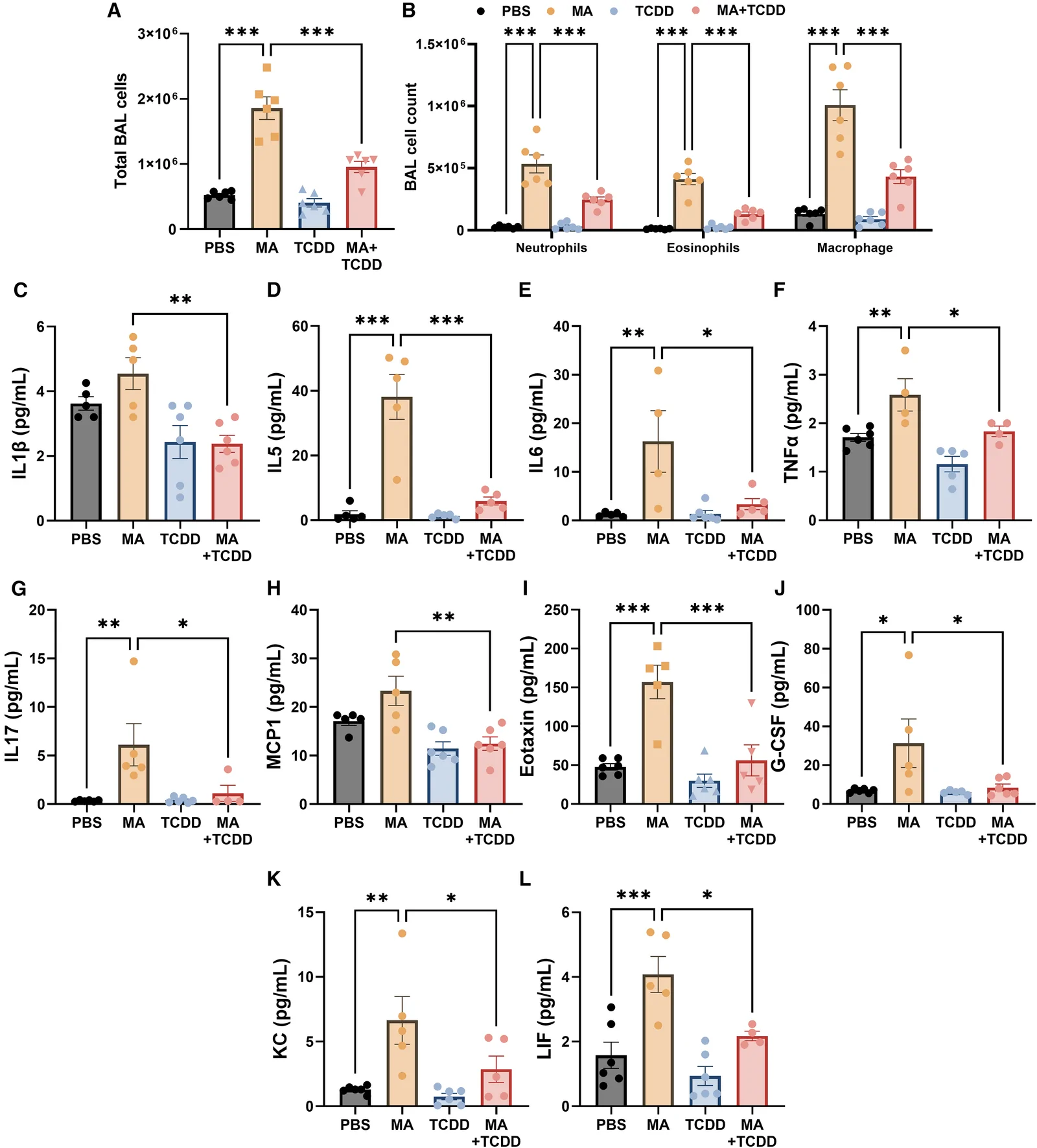
AhR activation abrogates immune cell infiltration and inflammatory cytokine levels in the BALF of MA-induced asthmatic mice MA-challenged mice exhibited significant increases in (A) total BAL cells and (B) leukocytes: neutrophils, eosinophils, and macrophages, (C) IL-1β, (D) IL-5, (E) IL-6, (F) TNF-α, (G) IL-17, (H) monocyte chemoattractant protein-1 (MCP-1), (I) eotaxin, (J) granulocyte colony-stimulating factor (G-CSF), (K) keratinocyte-derived chemokine (KC), and (L) leukemia inhibitory factor (LIF) compared with PBS controls. TCDD treatment alone did not significantly affect cytokine levels, whereas TCDD administration in MA-challenged mice markedly reduced the MA-induced BALF cytokine levels. Data were evaluated using one-way ANOVA with Tukey’s post hoc test for multiple comparisons. Mean ± SEM (*n* = 6 mice/group); ∗*p* < 0.05, ∗∗*p* < 0.01, ∗∗∗*p* < 0.001.

### AhR activation mitigates airway remodeling in MA-induced asthmatic mice

To correlate the findings of lung mechanics and cytokine profiles with structural changes in the airways, histological analyses were performed on H&E (Figure 3A), Masson’s trichrome (Figure 3B), and periodic acid-Schiff (PAS)-stained lung sections (Figure 3C). H&E-stained lung sections from MA-challenged mice showed a significant increase in airway wall thickness (Figures 3A and 3D) and inflammatory cell infiltration (Figures 3A and 3E) compared with PBS controls. Whereas TCDD treatment in MA-challenged mice significantly reduced both airway wall thickness and inflammatory cell recruitment, Masson’s trichrome staining revealed enhanced collagen deposition around the airways in MA-challenged mice compared to PBS controls (Figures 3B and 3F); whereas TCDD treatment significantly attenuated collagen deposition. Additionally, mucin production in airway epithelial cells, assessed by PAS staining (represented as PAS positive cells), was markedly elevated in MA-challenged mice compared with PBS counterparts (Figures 3C and 3G). TCDD administration substantially reduced MA-induced mucin production, further supporting the role of AhR activation in mitigating airway remodeling in asthma.

**Figure 3:**
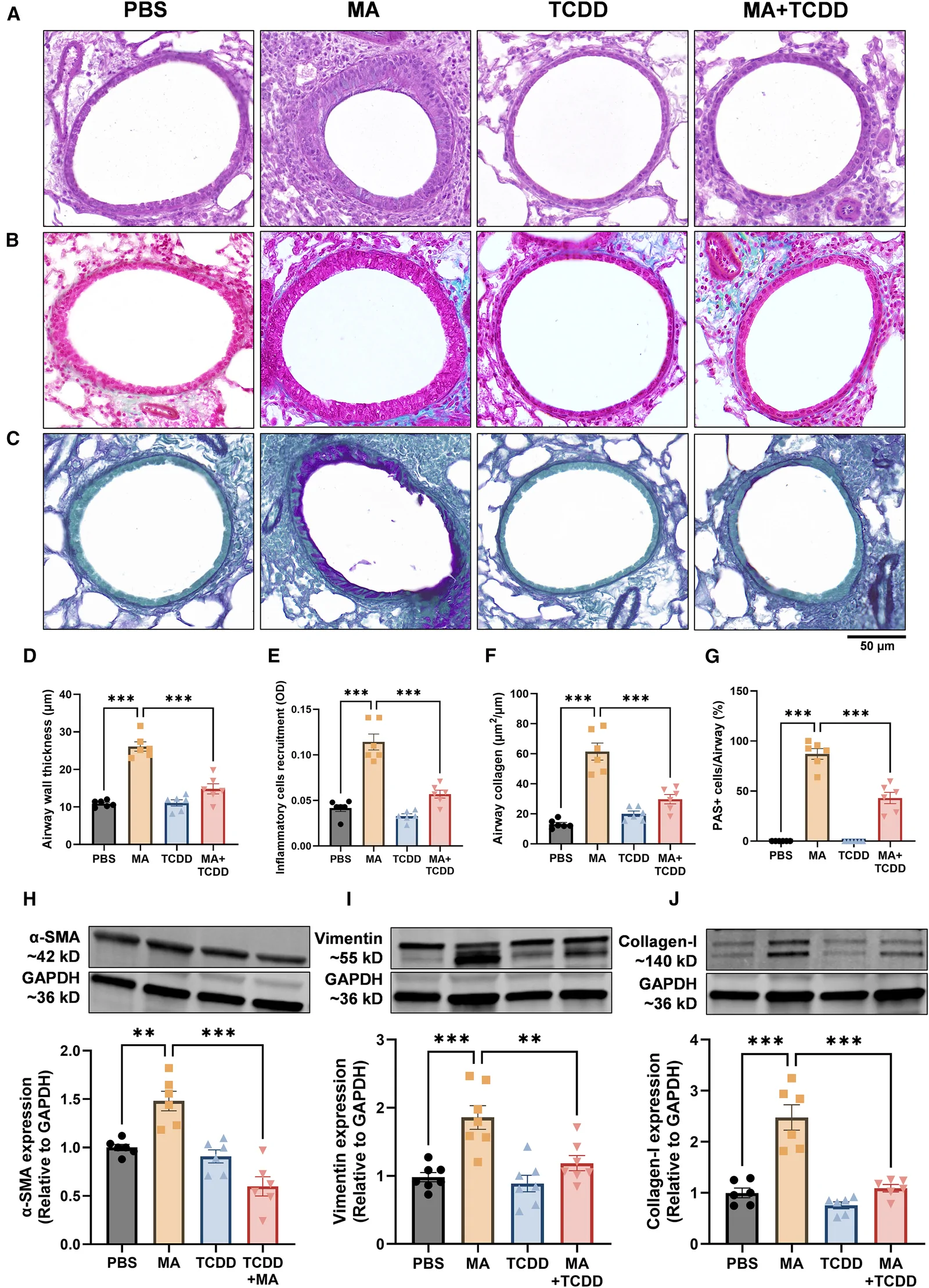
AhR activation mitigates airway remodeling in MA-induced asthmatic mice Lung sections were stained with (A) hematoxylin and eosin (H&E), (B) Masson’s trichrome, and (C) periodic acid-Schiff (PAS). (D–G) Quantitative analysis of airway wall thickness, inflammatory cell recruitment, collagen deposition, and mucin production. MA-challenged mice showed substantial increases in airway wall thickness, inflammatory cell infiltration, collagen deposition, and mucin production compared with PBS control. In contrast, TCDD-treated MA-challenged mice exhibited significant reductions in all these structural and inflammatory changes. Representative western blot images and quantitative densitometric analyses of (H) α-smooth muscle actin (α-SMA), (I) vimentin, and (J) collagen-I in whole lung tissue homogenates. MA-challenged mice showed significant upregulation of all three proteins compared with PBS control. TCDD treatment alone did not alter protein expression, whereas TCDD administration in MA-challenged mice significantly reduced α-SMA, vimentin, and collagen-I expression levels. Data were evaluated using one-way ANOVA with Tukey’s post hoc test for multiple comparisons. Mean ± SEM (*n* = 6 mice/group); ∗∗*p* < 0.01, ∗∗∗*p* < 0.001. Scale bars, 50 μm.

To further examine the molecular basis of AhR-mediated protection against airway remodeling, protein expression of important remodeling markers were investigated in lung homogenates. MA-challenged mice exhibited a significant increase in α-smooth muscle actin (α-SMA) (a marker of ASM hypertrophy) (Figure 3H); vimentin (cytoskeletal protein associated with mesenchymal phenotype transition and fibrosis) (Figure 3I); and collagen-I (major ECM component) (Figure 3J), compared with PBS-challenged controls. Treatment with TCDD alone did not alter the expression of these proteins compared to PBS controls. Notably, TCDD administration in MA-challenged mice significantly downregulated α-SMA, vimentin, and collagen-I expression, confirming that AhR activation attenuates airway remodeling at the molecular level as well. To further determine whether AhR activation modulates ASM remodeling, quantitative reverse-transcription PCR (RT-qPCR) was performed on RNA isolated from laser capture microdissected ASM. MA challenge increased the expression of remodeling-associated genes, including *Acta2*, *Col1a1*, *Vim*, and *Fn1*, whereas AhR activation significantly attenuated their expression (Figure S2). These findings further support an anti-remodeling effect of AhR signaling within the ASM compartment.

### AhR activation inhibits airway contractility in MA-induced asthmatic mice

To determine whether AhR activation influences airway contractility, precision-cut lung slices (PCLS) were produced from mice exposed to either PBS or MA (Figures 4A and 4B). PCLS from MA-challenged mice exhibited a significant increase in airway narrowing in response to increasing concentrations of MCh (1 nM–1 mM) compared with the PBS group, indicating heightened contractile sensitivity. Notably, treatment with TCDD significantly attenuated MCh-induced airway narrowing in MA-exposed mice (Figure 4C), demonstrating that AhR activation effectively reduces airway contractility. Analysis of AUC further confirmed a reduction in overall MCh-induced contractile responses in TCDD-treated asthmatic mice (Figure 4D).

**Figure 4:**
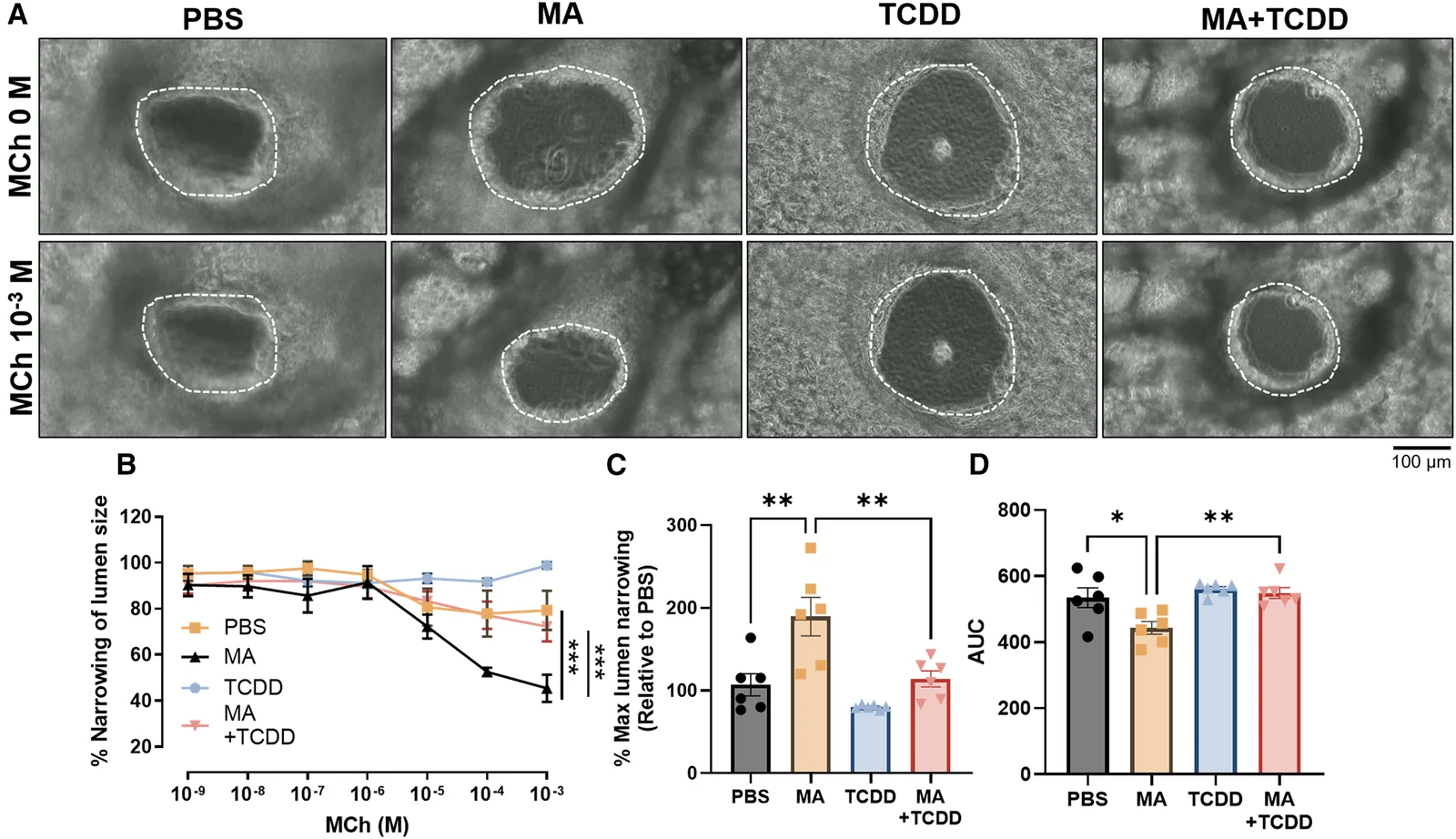
AhR activation reduces airway contractility in MA-induced asthmatic mice (A) Representative images of precision-cut lung slices (PCLS) prepared from PBS- and MA-exposed mice, with or without TCDD treatment. (B) Quantification of methacholine (MCh)-induced airway narrowing with increasing MCh concentrations (1 nM–1 mM). (C) Percentage of max airway narrowing in response to max concentration of MCh. (D) AUC. PCLS from MA-challenged mice exhibited significantly enhanced airway constriction compared with PBS controls. TCDD treatment markedly attenuated MCh-induced airway narrowing in MA-exposed mice, demonstrating that AhR activation reduces airway hypercontractility. Data were evaluated using repeated measures one/two-way ANOVA with Tukey’s post hoc test for multiple comparisons. Mean ± SEM (*n* = 6 mice/group); ∗*p* < 0.05, ∗∗*p* < 0.01. Scale bars, 100 μm.

### AhR is upregulated in the MA-induced asthmatic mice

To determine the effect of MA exposure on AhR expression, we evaluated AhR expression in whole lungs and in the isolated ASM layer (laser capture microdissected). Immunofluorescence analysis revealed localization and significantly higher AhR expression in the ASM of MA-challenged mice compared with PBS controls (Figures 5A and 5B). Consistently, western blot and qPCR analyses in lungs demonstrated significant upregulation of AhR mRNA (Figure 5C) and protein (Figure 5D) expressions in the MA-challenged mice compared to PBS. Interestingly, analysis of the isolated ASM layer confirmed a similar trend, with elevated AhR expression in ASM from MA-challenged mice compared with PBS controls (Figure 5E). Notably, TCDD-treated mice exhibited enhanced expression of CYP1B1, a canonical downstream target of AhR (Figure 5F), confirming effective AhR activation in the ASM.

**Figure 5:**
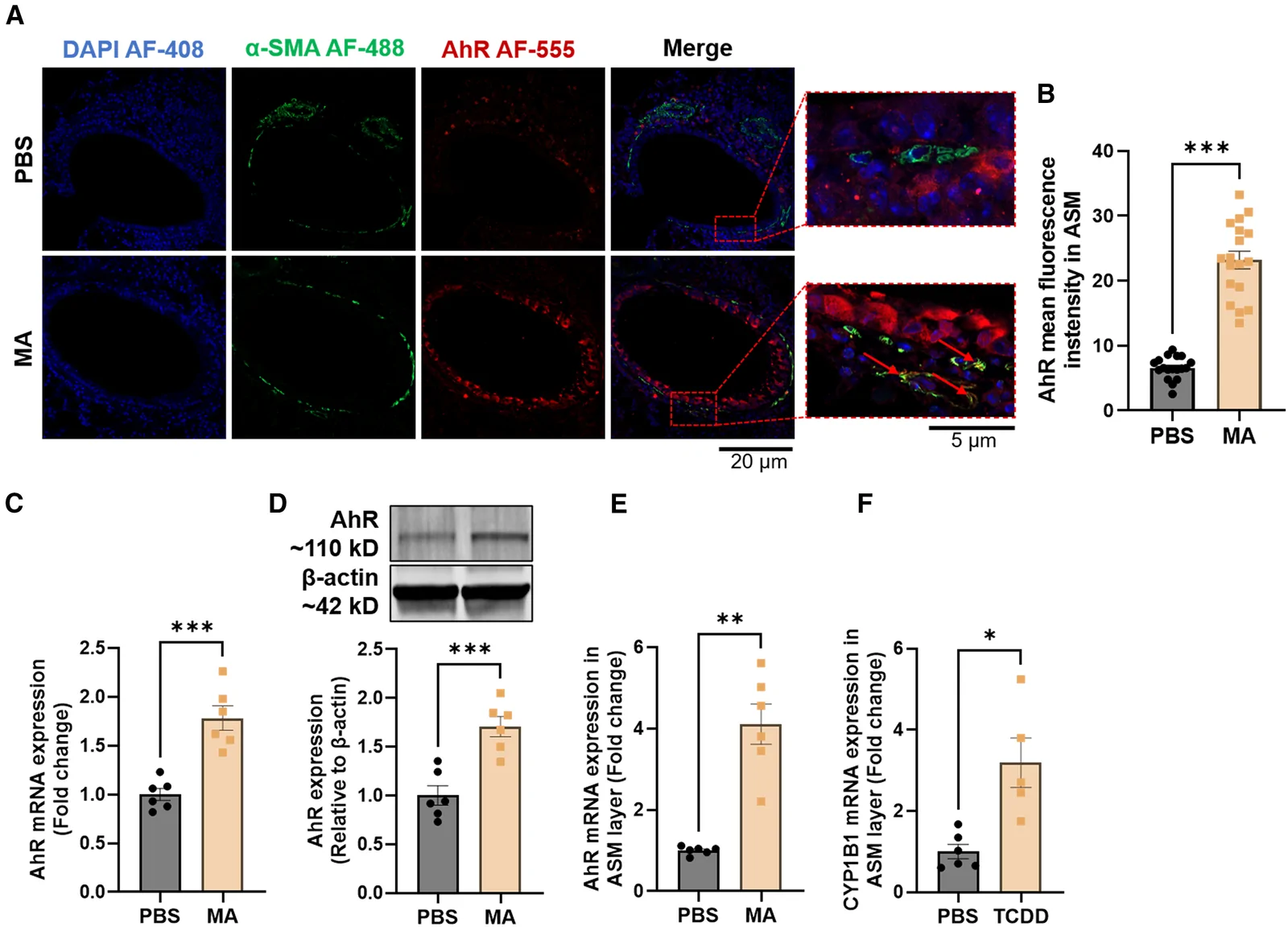
AhR is upregulated in MA-induced asthmatic mice (A and B) Immunofluorescence and corresponding quantitative analysis (MFI) of lung sections show localization and elevated AhR expression in the ASM layer of MA-challenged mice compared with PBS controls (MFI: 18 ROIs per group). (C and D) qPCR and western blot analyses demonstrated significant upregulation of AhR mRNA and protein expression, respectively, in lungs from MA-challenged mice. (E) Laser capture microdissection (LCM)-assisted isolated ASM layer demonstrated increased AhR expression in MA-challenged mice. (F) TCDD-treated mice showed enhanced expression of CYP1B1. Data were evaluated using unpaired Student’s *t* test. Mean ± SEM (*n* = 6 mice/group); ∗*p* < 0.05, ∗∗*p* < 0.01, ∗∗∗*p* < 0.001. Scale bars, 20 μm and 5 μm (magnified regions).

### AhR activation attenuates MA-induced inflammatory transcriptional changes

To determine the transcriptional changes induced by MA exposure and AhR activation, differential gene expression analysis was performed on bulk RNA sequencing (RNA-seq) data of lungs in three comparisons: MA vs. PBS, TCDD vs. PBS, and MA + TCDD vs. MA. Differentially expressed genes (DEGs) were visualized using volcano plots and corresponding heatmaps of the top 50 DEGs (Figure 6).

**Figure 6:**
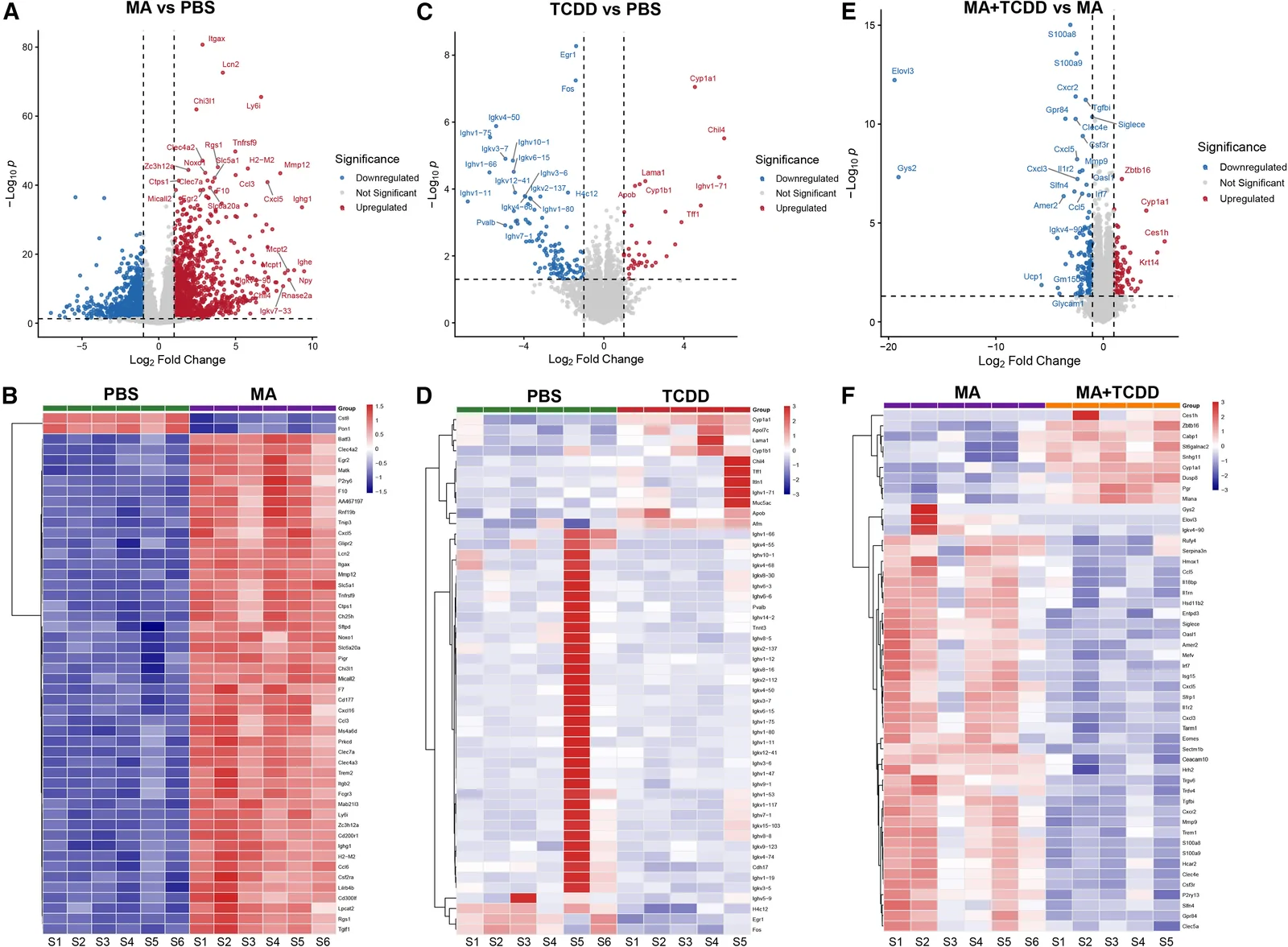
Differential gene expression analysis reveals inflammatory transcriptional changes in the lungs Volcano plots and heatmaps illustrate differentially expressed genes (DEGs) across three comparisons: (A and B) MA vs. PBS, (C and D) TCDD vs. PBS, and (E and F) MA + TCDD vs. MA. Volcano plots display log_2_ fold change versus −log10 *p* value, with significantly regulated genes highlighted. Red points indicate upregulated genes (log2FC ≥ 1, *p* < 0.05), blue points indicate downregulated genes (log2FC ≤ −1, *p* < 0.05), and gray points indicate non-significant genes. Dashed lines indicate significance cutoffs (horizontal: *p* = 0.05; vertical:|log2FC| = 1). Corresponding heatmaps show the top 50 DEGs based on *p* value. Color intensity indicates row-normalized (*Z* score) expression levels, with red indicating higher expression and blue indicating lower expression relative to the row mean. MA challenge induced robust upregulation of inflammatory, immune, and remodeling-associated genes, whereas TCDD treatment alone selectively induced AhR target genes (Cyp1a1, Cyp1b1). In MA-challenged mice, TCDD treatment substantially suppressed various inflammatory and neutrophil-associated genes. *n* = 5–6 mice/group.

MA-challenged mice revealed extensive transcriptional reprogramming consistent with airway inflammation (Figures 6A and 6B). MA exposure resulted in marked upregulation of genes associated with immune cell recruitment, inflammation, and airway remodeling, including *Cxcl5*, *Ccl3*, *Mmp12*, *Chi3l1*, *Chil4*, *Rnase2a*, and mast cell-associated proteases (*Mcpt1*, *Mcpt2*). Furthermore, elevation of immunoglobulin genes (*Ighe*, *Ighg1*, *Igkv* family members) indicated enhanced adaptive immune activation. Collectively, these transcriptional changes confirm successful induction of asthmatic phenotype by MA challenge. Interestingly, MA exposure alone did not induce the canonical AhR target genes *Cyp1a1*, *Cyp1b1*, *Ahrr*, or *Nqo1* (Figure S4), indicating that allergen challenge does not activate canonical AhR transcriptional signaling. In contrast, TCDD treatment alone produced limited transcriptional change. Notably, conventional AhR target genes *Cyp1a1*, *Cyp1b1*, *Ahrr*, and *Nqo1* were significantly upregulated, confirming effective AhR activation in the lung (Figures 6C, 6D, and S4). On the other hand, immediate-early genes (*Egr1*, *Fos*) and multiple immunoglobulin genes were downregulated with TCDD exposure, indicating that AhR activation in the absence of allergen challenge does not promote pro-inflammatory genes. MA-challenged mice treated with TCDD revealed markedly attenuated MA-induced inflammatory gene expression (Figures 6E and 6F). Moreover, genes associated with neutrophil recruitment and activation, including *S100a8*, *S100a9*, *Cxcr2*, *Cxcl3*, *Cxcl5*, *Csf3r*, *Mmp9*, and *Gpr84*, were significantly downregulated following TCDD treatment. Inflammatory signaling mediators such as *Il1r2* and *Tgfbi* were also reduced. Importantly, canonical AhR target genes expression remained upregulated in MA + TCDD lungs, confirming sustained AhR activation (Figures 6E, 6F, and S5). Together, these data indicate that AhR activation inhibits MA-induced inflammatory transcriptional changes.

### AhR activation reverses MA-induced inflammatory and remodeling pathways

To identify biological pathways associated with MA-induced asthma and the functional changes induced by AhR activation, Ingenuity Pathway Analysis (IPA) was performed using DEGs (Figure 7). IPA revealed significant enrichment of multiple inflammatory and immune-related canonical pathways in MA-challenged lungs compared with PBS controls (Figure 7A; Table S1). These included Th2, Th1-, and innate immune pathways such as IL-4/IL-13 signaling, TNF receptor signaling, interferon-γ signaling, and Toll-like receptor cascades. Further, pathways involved in oxidative stress such as Rho GTPase-mediated activation of nicotinamide adenine dinucleotide phosphate (NADPH) oxidases, were also activated. Importantly, MA exposed mice showed robustly enriched pathways associated with airway remodeling, including collagen degradation, ECM degradation, integrin cell surface interactions, and matrix metalloproteinase activation, strengthening the histological and protein-level evidence of airway remodeling. Whereas TCDD treatment in MA-challenged mice significantly suppressed inflammatory and immune-related pathways (Figure 7B; Table S2), including IL-4/IL-13 signaling, interferon-γ signaling, interferon α/β signaling, Gα(i) signaling, TLR regulation by endogenous ligands and IL-1 family signaling. In addition, genes associated with neutrophil activation (*S100a8*, *S100a9*) and chemokine signaling (*Cxcr2*, *Ccl5*) were also downregulated in MA + TCDD mice. These findings demonstrate that AhR activation reprograms MA-induced inflammatory networks toward a suppressed, anti-inflammatory state.

**Figure 7:**
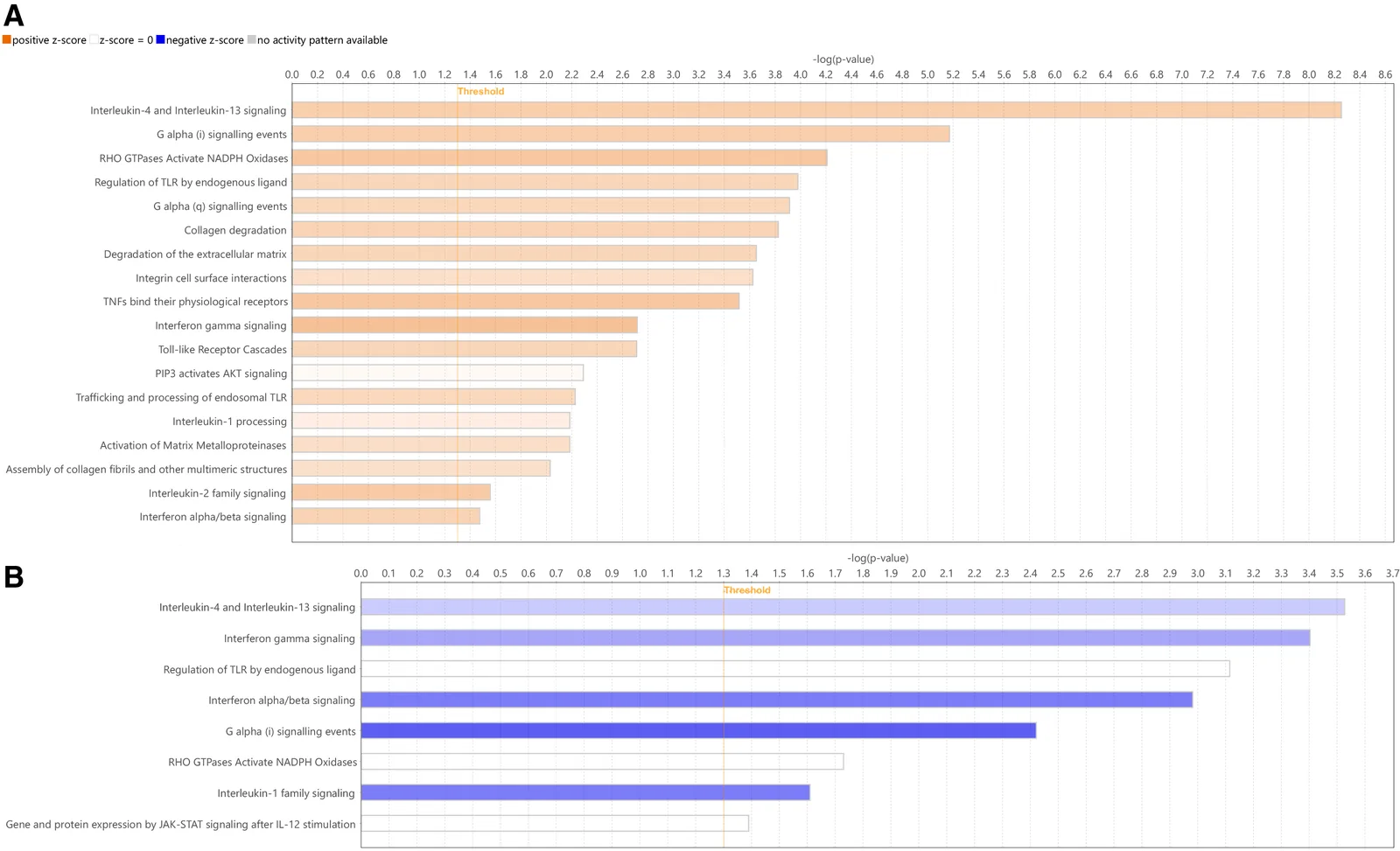
Ingenuity pathway analysis reveals suppression of inflammatory and remodeling pathways by AhR activation (A) Canonical pathways enriched in PBS vs. MA comparison demonstrate activation of inflammatory, immune, oxidative stress, and ECM remodeling pathways following MA challenge. (B) Canonical pathways enriched in MA + TCDD vs. MA comparison show suppression of inflammatory and innate immune signaling pathways following AhR activation. Pathway significance is represented by −log(*p* value), and predicted activation states are indicated by *Z* scores. A positive *Z* score indicates pathway is likely activated, while a negative *Z* score indicates it is likely inhibited. *n* = 5–6 mice/group.

### Integrated upstream and downstream network analysis reveals AhR-mediated suppression of inflammatory networks in MA-induced asthma

To delineate regulatory drivers and transcriptional consequences underlying MA-induced asthma and their modulation by AhR activation, integrated upstream and downstream network analyses were performed using IPA (Figure 8).

**Figure 8:**
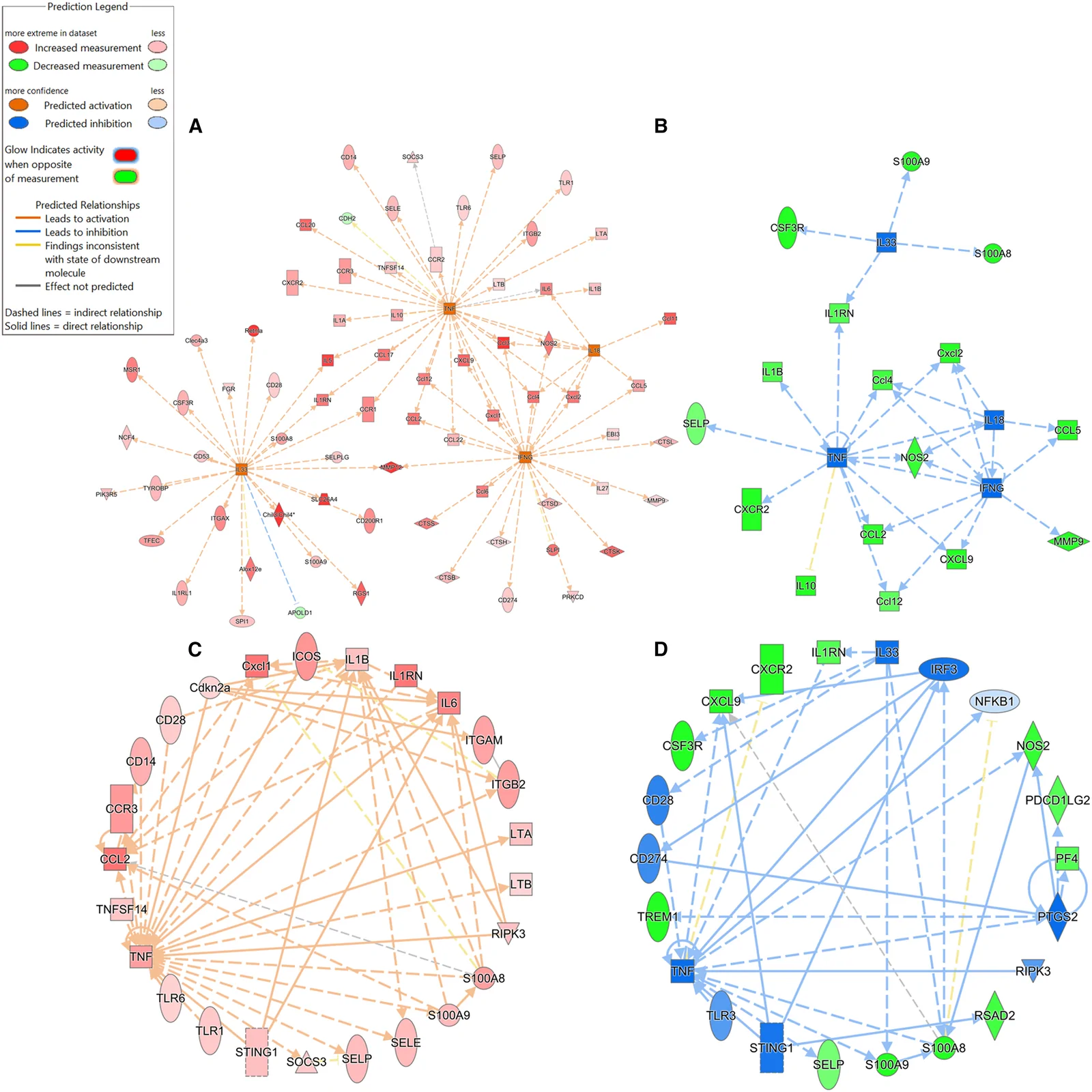
Integrated upstream and downstream network analysis reveals AhR-mediated suppression of inflammatory regulatory networks in MA-induced asthma Ingenuity pathway analysis (IPA)-derived networks are shown for upstream regulators and downstream effector genes. (A) Upstream regulatory network activated in PBS vs. MA comparison. (B) Suppressed upstream regulatory network in MA + TCDD vs. MA comparison. (C) Downstream effector network activated in PBS vs. MA comparison. (D) Downstream effector network suppressed following AhR activation in MA + TCDD vs. MA comparison. *n* = 5–6 mice/group.

Upstream network analysis of MA vs. PBS revealed robust activation of inflammatory and immune regulatory networks (Figure 8A). MA exposure induced strong expressions of chemokines and cytokines involved in leukocyte recruitment and activation, including *Ccl2*, *Ccl3*, *Ccl4*, *Ccl5*, *Ccl11*, *Ccl12*, *Ccl17*, *Ccl20*, *Ccl22*, *TNF*, *IFNG*, *IL1A*, *IL1B*, *IL5*, *IL6*, and *IL33*. These upstream signals were accompanied by increased expression of immune receptors and adhesion molecules (*CCR1*, *CCR2*, *CCR3*, *CXCR2*, *ITGAX*, *ITGB2*, and *CD14*), indicating enhanced immune cell trafficking and activation. Consistent with upstream networks, downstream network analysis showed coordinated induction of pro-inflammatory genes (Figure 8C). Furthermore, genes associated with innate immune signaling (*TLR1*, *TLR6*, *STING1*), cytokine signaling (*IL1B*, *IL6*, *TNF*), leukocyte adhesion (*SELE*, *SELP*), and neutrophil activation (*S100A8*, S100A9) were significantly upregulated. In addition, immune costimulatory and signaling molecules (*CD28*, *ICOS*, *SOCS3*) and ECM-associated mediators (*ITGAM*, *ITGB2*) were elevated, indicating downstream activation of MA-induced inflammatory signals. Together, these upstream and downstream network analyses indicate a highly coordinated inflammatory regulatory network inducing airway inflammation and remodeling in MA-induced asthma.

In contrast, AhR activation with TCDD in MA-challenged mice resulted in suppression of both upstream regulatory signals and downstream inflammatory responses (Figures 8B and 8D). Upstream network analysis revealed significant downregulation of key chemokines (*Ccl2*, *Ccl4*, *Ccl5*, *Ccl12*, *Cxcl2*, *Cxcl9*) and cytokines (*TNF*, *IFNG*, *IL1B*, *IL10*), along with reduced expression of neutrophil- and myeloid-associated genes (*CSF3R,* S100A8, S100A9*, NOS2*). Reduction of *CXCR2* and *SELP* further suggests attenuated inflammatory cell recruitment. Subsequently, downstream network analysis demonstrated coordinated inhibition of pro-inflammatory pathways. Genes involved in innate immune signaling (*STING1*, *TLR3*), cytokine and chemokine signaling (*TNF*, *CXCL9*, *CXCR2*), immune checkpoint regulation (*CD274*, *PDCD1LG2*), and inflammatory mediators (*PTGS2*, *TREM1*) were reduced following TCDD treatment. Notably, strong downregulation of neutrophil-associated genes (*S100A8*, S100A9) indicates effective suppression of innate inflammatory responses. Collectively, these findings demonstrate that AhR activation rewires MA-induced regulatory networks by reducing both upstream inflammatory drivers and downstream effector responses, providing a mechanistic basis for the observed improvements in airway inflammation, hyperresponsiveness, and remodeling.

## Discussion

In this study, we provide comprehensive *in vivo* evidence that pharmacological AhR activation with TCDD significantly attenuates airway hyperresponsiveness, inflammation, and remodeling in a chronic MA-induced mouse model of asthma. By integrating lung function, PCLS functional assays, laser-capture microdissection (LCM), and global transcriptomic profiling with pathway and network interrogation, our findings establish AhR as a central regulator of both inflammatory and structural components of asthma pathophysiology.

The MA model used in this study most closely models severe allergic asthma characterized by persistent airway hyperresponsiveness, chronic airway inflammation, and structural remodeling.^33^^,^^34^^,^^35^ In addition to robust eosinophilic inflammation, the induction of neutrophil-associated cytokines and chemokines as observed in our transcriptomic and BALF analyses suggests that this model also recapitulates important features of mixed granulocytic asthma, a phenotype frequently associated with greater disease severity and airway remodeling.^36^ Although corticosteroid responsiveness was not evaluated in the present study, the persistent inflammatory and remodeling phenotype shares characteristics commonly associated with difficult-to-treat asthma.^37^

Chronic MA exposure produced robust airway hyperresponsiveness characterized by increased Rrs and Ers with reduced Crs, similar to major functional abnormalities observed in human asthma.^2^^,^^38^^,^^39^ These findings are consistent with previous studies using MA models that better recapitulate the heterogeneity and severity of clinical asthma compared to single-allergen-induced asthma.^40^ Notably, activation of AhR with TCDD significantly restored lung function in MA-challenged mice without affecting baseline lung function in control animals, suggesting that AhR signaling selectively counteracts asthma-associated abnormalities. These observations align well with earlier work showing protective AhR functions in other airway structural cells. For example, AhR activation in airway epithelial cells has been shown to limit mucus production, dampen inflammation, and reduce remodeling in mouse models of asthma.^23^^,^^41^

The protective effects of AhR activation were further supported by significant suppression of leukocyte infiltration and inflammatory cytokine production in BALF. MA challenge induced a broad inflammatory milieu including Th2 (IL-5, IL-13, eotaxin), Th17 (IL-17), and innate immune (IL-1β, TNF-α, KC, G-CSF) cytokines, reflecting the complex immune environment of chronic asthma. Interestingly, TCDD treatment substantially reduced all measured cytokines and immune cell infiltration, consistent with previous studies demonstrating that AhR activation prevents excessive immune activation by modulating dendritic cell function, T cell polarization, and innate immune signaling.^42^^,^^43^ Moreover, these findings align with a study from AhR-deficient model, which show exaggerated airway inflammation following allergen exposure,^44^ strengthening the endogenous anti-inflammatory role of AhR signaling in the lung.

Airway remodeling is a critical mediator of persistent airflow obstruction in asthma and remains poorly responsive to current anti-inflammatory therapies.^1^^,^^9^ Here, MA-challenged mice exhibited hallmark features of remodeling, including increased airway wall thickness, collagen deposition, mucin production, and elevated expression of α-SMA, vimentin, and collagen-I. AhR activation significantly attenuated all these structural changes. These findings are consistent with emerging evidence that AhR signaling attenuates fibroblast activation, limits ECM production, and inhibits profibrotic signaling pathways such as TGF-β.^45^^,^^46^ The ability of AhR activation to simultaneously control inflammation and remodeling in asthma model demonstrates its unique therapeutic potential, as these pathologies are tightly interlinked yet often poorly addressed by existing therapies. Future studies employing delayed administration of selective AhR agonists after establishment of chronic asthma will be important to determine the therapeutic potential of targeting AhR signaling in asthma.

An important strength of this study is the incorporation of PCLS-based functional assays, which provide direct insight into airway contractility independent of systemic influences.^47^ MA-derived *ex vivo* lung slices displayed exaggerated MCh-induced airway narrowing, confirming intrinsic airway hypercontractility. On the other hand, TCDD treatment in MA-challenged mice significantly reduced this contractile response, demonstrating an association between pharmacological AhR activation and reduced-ASM hypercontractility during asthma. These findings complement our earlier *in vitro* work showing that AhR activation stabilizes ASM phenotype by inhibiting inflammation-induced mitochondrial fission via transcriptional repression of DRP1,^31^^,^^32^ and suppressing proliferative^24^ and remodeling^26^ responses. Consistent with these observations, RT-qPCR analysis of laser capture microdissected ASM further demonstrated that AhR activation attenuated the expression of several remodeling-associated genes, including *Acta2*, *Col1a1*, *Vim*, and *Fn1* (Figure S2), providing additional molecular evidence that AhR activation is associated with reduced remodeling-associated gene expression within the ASM compartment *in vivo.*

Consistent with our previous studies in human ASM cells,^25^^,^^27^ we observed a marked upregulation of AhR expression in MA-challenged lungs, including within the ASM layer isolated by LCM. Importantly, our previous *in vitro* studies demonstrated that although inflammatory stimulation and asthmatic ASM increased AhR expression, canonical AhR transcriptional activity remained low unless the receptor was activated by an exogenous ligand.^27^^,^^31^ Consistent with these observations, MA exposure alone failed to induce, and in fact modestly reduced, the expression of canonical AhR target genes (*Cyp1a1*, *Cyp1b1*, *Ahrr*, and *Nqo1*) despite increased AhR expression (Figure S4), indicating that inflammatory allergen challenge elevates AhR expression without altering ligand-dependent transcriptional activity. We interpret this pattern as a compensatory but functionally inactive endogenous response, in which AhR expression is induced during chronic inflammation yet remains inactive to counteract airway inflammation and remodeling. Whereas pharmacologic AhR activation with TCDD led to strong induction of classical AhR target genes, including *Cyp1a1* and *Cyp1b1,* confirming effective receptor activation *in vivo* and demonstrating that the receptor remains functionally competent when appropriately ligated. Importantly, this finding aligns with our recent findings in human ASM cells, where AhR overexpression alone failed to modulate inflammation-induced mitochondrial fission, while ligand-mediated AhR activation effectively inhibited this pathological process.^31^ Together, these findings demonstrate that AhR expression and AhR activation are biologically distinct processes: asthma upregulates receptor expression as a compensatory response, but insufficient endogenous ligand-dependent activation limits its protective capacity, whereas pharmacological AhR activation with TCDD restores canonical signaling and suppresses asthma-associated inflammation and remodeling.

Bulk RNA-seq of lungs further strengthened these mechanistic insights. MA challenge induced upregulation of genes associated with immune cell recruitment, neutrophil activation, mast cell responses, and tissue remodeling. In contrast, TCDD treatment in MA-challenged mice suppressed inflammatory and neutrophil-associated genes while preserving canonical AhR signaling. Furthermore, IPA-based pathway analysis revealed strong activation of IL-4/IL-13, TNF, interferon, Toll-like receptor, and ECM remodeling pathways in MA lungs, all of which were inhibited with AhR activation. Interestingly, integrated upstream and downstream network analyses demonstrated coordinated suppression of inflammatory regulatory networks, including chemokine-cytokine axes and innate and adaptive immune signaling nodes, providing a systems-level explanation for the observed physiological and structural improvements with AhR activation.

Overall, these findings support a regulatory role for AhR signaling in airway inflammation and remodeling. Pharmacological activation with TCDD was associated with suppression of inflammation and remodeling and improvement in airway function.

In conclusion, this study supports AhR signaling as a critical regulator of airway inflammation, remodeling, and hyperresponsiveness in experimental asthma. Using integrated physiological, structural, and transcriptomic analyses, we demonstrate that MA exposure induces robust airway hyperresponsiveness and remodeling while increasing AhR expression in the lung and ASM without effectively activating AhR pathway. Pharmacological activation of AhR with TCDD restores functional signaling, suppresses inflammatory, and remodeling-associated gene networks, reduces ASM-driven hypercontractility, and improves lung function. Future studies incorporating cell-specific and ligand-selective approaches will be important to further define the translational potential of targeting AhR signaling in asthma.

### Limitations of the study

Some limitations of the present study should be acknowledged. First, differential analyses were performed using *p* value thresholds rather than false discovery rate (FDR)-adjusted *p* values, which increases the likelihood of false-positive gene identification. Albeit, the key pathways and networks identified in IPA, particularly those related to inflammatory signaling, neutrophil recruitment, airway remodeling, and AhR signaling were highly coherent, directionally consistent, and aligned with robust physiological, histological, and functional outcomes, mitigating concerns that the conclusions are driven by spurious transcript-level changes. Nevertheless, future studies with larger sample sizes and FDR-controlled analyses will further strengthen these findings. Second, transcriptomic profiling was performed on whole lung tissue, which demonstrates a composite signal from multiple cell types in lungs. To partially address this limitation, we complemented bulk RNA- seq with laser capture microdissection of the ASM layer and functional PCLS assays, which together provide spatial and physiological context for ASM-specific effects. However, future studies using single-cell or spatial transcriptomic approaches will be important to resolve cell-type ASM-specific AhR-dependent regulatory mechanisms in more detail. Third, TCDD was used as a prototypical high-affinity AhR agonist to establish mechanistic proof-of-concept. While TCDD provides sustained AhR activation, its toxicological properties preclude direct clinical translation to human asthma. Accordingly, the present findings should be interpreted as evidence supporting the protective effects of AhR activation rather than the therapeutic use of TCDD itself. Although formal systemic toxicity assessments were not performed, no treatment-related mortality or overt clinical signs of toxicity were observed in mice during the study. Future studies should evaluate endogenous or selective AhR agonists with improved safety profiles, such as 6-formylindolo[3,2-b]carbazole (FICZ) or 2-(1′H-indole-3′-carbonyl)-thiazole-4-carboxylic acid methyl ester (ITE), while incorporating comprehensive toxicological evaluation to establish translational feasibility. Lastly, although TCDD induced canonical AhR target genes in this study, genetic loss of function approaches was not employed; therefore, definitive attribution of the observed effects to AhR will require future validation using ASM-specific AhR knockout models.

## Resource availability

### Lead contact

Further information and requests for resources and reagents should be directed to and will be fulfilled by the lead contact, Venkatachalem Sathish (sathish-venkatachalem@ou.edu).

### Materials availability

This study did not generate new unique reagents.

### Data and code availability

• Raw RNA-seq data generated in this study are publicly available in the NCBI Gene Expression Omnibus as GEO: GSE325010. All other data supporting the findings of this study are available within the article and its supplemental information.
• This paper does not report original code.
• Any additional information required to reanalyze the data reported in this paper is available from the lead contact upon request.

## Acknowledgments

This study was supported by 10.13039/100000002NIH grants: R01-HL171245 (to V.S. and R.D.B.), R01-HL155095 (to R.D.B.). The graphical abstract was created using a licensed version of BioRender.

## Author contributions

M.I.R., R.D.B., Jr., and V.S. conceptualized and designed the study including experimental details and protocols. M.I.R., P.B., A.K., K.M.H., and J.W. performed experiments, collected, and analyzed the data. M.I.R., P.B., A.K., K.M.H., J.W., R.D.B., Jr., and V.S. designed and finalized the data and figures. M.I.R. wrote the initial drafts of the manuscript. M.I.R. and V.S. collated suggestions and edits from all authors for the final version of the manuscript and figures. All authors approved the final submission.

## Declaration of interests

The authors declare no competing interests.

## STAR★Methods

### Key resources table

**Table:** 

REAGENT or RESOURCE	SOURCE	IDENTIFIER
**Antibodies**		

Anti-AhR Antibody	Invitrogen	Cat# MA1-514; RRID:AB_2273723
Anti-AhR Antibody	Abcam	Cat# ab308215; RRID:AB_3083080
Anti-α-SMA Antibody	Millipore Sigma	Cat# A2547; RRID:AB_476701
Anti-α-SMA Antibody	Cell Signaling Technology	Cat# 19245S; RRID:AB_2734735
Anti-Vimentin Antibody	Millipore Sigma	Cat# V5255; RRID:AB_477625
Anti-Collagen-I Antibody	Abcam	Cat# ab260043; RRID:AB_2922767
Anti-β-actin Antibody	Applied Biological Materials	Cat# G043; RRID:AB_2631287
Anti-GAPDH Antibody	Cell Signaling Technology	Cat# 5174; RRID:AB_10622025
Donkey anti-Rabbit IgG H + L Highly Cross Adsorbed Alexa Fluor 488	Invitrogen	Cat# A-21206; RRID: AB_2535792
Donkey anti-Mouse IgG H + L Highly Cross Adsorbed Alexa Fluor 555	Invitrogen	Cat# A-31570; RRID: AB_2536180
IRDye 800CW Goat anti-Rabbit	LI-COR Biosciences	Cat# 926–32211; RRID AB_621843
IRDye 680LT Goat anti-Mouse	LI-COR Biosciences	Cat# 926–68020; RRID AB_10706161

**Chemicals, peptides, and recombinant proteins**		

TCDD	AccuStandard	Cat# D-404N
*Alternaria alternata*	Greer Laboratories	Cat# XPM1D3A2.5
*Aspergillus fumigatus*	Greer Laboratories	Cat# XPM3D3A4
*Dermatophagoides pteronyssinus*	Greer Laboratories	Cat# XPB91D3A25
Ovalbumin	InvivoGen	Cat# vac-pova-25

**Deposited data**		

Public RNA-seq dataset	Gene Expression Omnibus	GEO: GSE325010

**Experimental models: Organisms/strains**		

Mouse: C57BL/6J	Jackson Laboratory	Strain# 000664

**Oligonucleotides**		

*Ahr* primers	This paper	Forward: AGAGCATTTACAGAAGCGAA; Reverse: AGTCTTTCCTGCTAGTGTTG
*Cyp1b1* primers	This paper	Forward: AGGCTGCAAGACAGAGAAAG; Reverse: ACTGACACAACCTGCGTATC
*Acta2* primers	This paper	Forward: GTGGCTATTCCTTCGTGACTAC; Reverse: AGCTACATAGCACAGCTTCTC
*Myh11* primers	This paper	Forward: CTATACCTACTCTGGCCTCTTCT; Reverse: GGCATCTCATGCCTCTTCTT
*Cdh1* primers	This paper	Forward: TTGGTGTGGGTCAGGAAATC; Reverse: GTGTCCCTCCAAATCCGATAC
*Ptprc* primers	This paper	Forward: CCCTTCTTCTGCCTCAAAGT; Reverse: CACCTGGATGATATGTGGTCTC
*Col1a1* primers	This paper	Forward: GCAAAGAGTCTACATGTCTAGGG; Reverse: GACTTCAGGGATGTCTTCTTGG
*Fn1* primers	This paper	Forward: ATGAGCGCCCTAAAGATTCC; Reverse: CAGCGATTTGCAATGGTACAG
*Vim* primers	This paper	Forward: CGGAAAGTGGAATCCTTGCAGG; Reverse: AGCAGTGAGGTCAGGCTTGGAA
*S16* primers	This paper	Forward: GCCCTGGTAGCTTATTAC; Reverse: CCGATCGTATTGGATGAG

**Software and algorithms**		

GraphPad Prism 10.5	GraphPad Software	https://www.graphpad.com/
ImageJ/Fiji	NIH	https://imagej.net/software/fiji/
Ingenuity Pathway Analysis	QIAGEN	https://digitalinsights.qiagen.com/products-overview/discovery-insights-portfolio/analysis-and-visualization/qiagen-ipa
R-Studio	Posit	https://posit.co/downloads
BioRender	BioRender	https://biorender.com/
Zeiss Zen 3.0	Zeiss	https://www.zeiss.com/microscopy/us/products/software/zeiss-zen.html

### Experimental model and study participant details

#### Experimental animals

All animal experiments were conducted in accordance with the National Institutes of Health Guide for the Care and Use of Laboratory Animals and were approved by the Institutional Animal Care and Use Committee (IACUC) at North Dakota State University (Ref# IACUC20240008).^48^ Wild-type (WT) C57BL/6J breeding pairs (male and female) were obtained from The Jackson Laboratory (Bar Harbor, ME, USA). In-house breeding was conducted, and offspring aged 6-8 weeks (20-25 g) were used for experiments. Animals were maintained under controlled environmental conditions (12 h light/dark cycle, constant temperature), with food and water provided *ad libitum*. Male and female mice were distributed approximately equally among experimental groups (3-4 animals of each sex per group, total n=7-8 per group): Vehicle (PBS-challenged), Mixed Allergen (MA)-challenged, TCDD-treated, and MA + TCDD-treated.

### Method details

#### Mixed allergen challenge and treatments

Male and female mice were intranasally challenged to a mixture of all four allergens (10 μg each, suspended in 25 μL PBS): ovalbumin (Protein: minimum 98%, Endotoxin: <1 EU/mg), *Alternaria alternata* (Protein: 2.02 mg/vial)*, Aspergillus fumigatus* (Protein: 2.32 mg/via), and *Dermatophagoides pteronyssinus* (Protein: 44.9 mg/vial, Endotoxin: 2000 EU/vial), on alternate days for a total duration of 4 weeks (3 days/week), as described previously.^33^ Control animals received 25 μL PBS following the same schedule. From day 2, the TCDD and MA + TCDD groups received TCDD (10 μg/kg)^49^ intranasally in 25 μL PBS on alternate days (3 days/week). All administrations were performed under isoflurane anesthesia. The experimental design is illustrated in Figure 1A.

#### Lung function analysis

On day 29, pulmonary mechanics were assessed using the flexiVent FX1 system (Scireq, Montreal, Canada). Mice were anesthetized using the cocktail of ketamine (100 mg/kg, i.p.) and xylazine (10 mg/kg, i.p.). Lung function parameters, including total respiratory system resistance (Rrs), elastance (Ers), and compliance (Crs), were assessed under baseline conditions (0 mg/mL methacholine, MCh) and following exposure to increasing concentrations of nebulized MCh (6.25-50 mg/mL). The maximum values at 50 mg/mL MCh were used for inter-group comparisons.^33^^,^^34^

#### Bronchoalveolar lavage fluid (BALF) processing and tissue collection

Following lung function assessment, BALF was collected by instilling 1.0 mL PBS containing protease inhibitors through a tracheal cannula, with approximately 600 μL recovered. Samples were centrifuged at 2,000 rpm for 5 min at 4°C, and the supernatant was stored at −80°C for cytokine analysis. Red blood cells were lysed using ACK Lysing Buffer (Thermo Fisher Scientific). BAL cells were stained with a Differential Quick Staining Kit (Modified Giemsa, EMS), and differential leukocyte counts (macrophages, eosinophils, neutrophils, and lymphocytes) were determined by counting a minimum of 100 cells per sample under light microscopy by an investigator blinded to the experimental groups. Separately, lungs were perfused with 10 mL PBS via the right ventricle to flush out residual blood. The left lobe was fixed in Carnoy’s solution (ethanol:chloroform:acetic acid, 6:3:1 with ferric chloride; Sigma-Aldrich) for several hours, followed by transfer to 70% ethanol for histological processing.^34^^,^^50^

#### Cytokine ELISA multiplex assay in BALF

BALF samples were centrifuged, aliquoted, and analyzed using a cytokine multiplex ELISA kit (Eve Technologies, Calgary, Canada), following the manufacturer’s instructions.^34^^,^^50^

#### Histopathology analyses

Paraffin-embedded left lung lobes were sectioned and stained with H&E (Sigma-Aldrich), PAS (EMS, Hatfield, PA, USA), and Masson’s Trichrome (Chondrex, Redmond, WA, USA), following standard procedures. We scanned the slides through an Epredia Pannoramic MIDI II (3DHISTECH, Budapest, Hungary). Quantitative analyses were performed using ImageJ (NIH, https://imagej.net/software/fiji/) by an investigator blinded to the treatment groups. For each mouse, 3-5 randomly selected intrapulmonary airways with a comparable basement membrane perimeter (approximately 50-100 μm) and near-circular morphology were analyzed from a single lung section. Airways adjacent to large blood vessels, collapsed airways, or incompletely sectioned airways were excluded from analysis. Airway inflammatory cell infiltration and wall thickness were quantified from H&E-stained sections, mucin production was quantified as the percentage of PAS-positive epithelial cells per airway, and collagen deposition was quantified from Masson's trichrome-stained sections and normalized to airway perimeter (μm^2^/μm) as previously described.^33^

#### Precision cut lung section (PCLS) study

PCLS and their contraction studies were performed based on established protocol.^34^ A separate cohort of mice was used for PCLS studies. After euthanasia with CO_2_ asphyxiation, lungs were inflated with 2% low-melting agarose prepared in HBSS (incubated at 42°C) and subsequently sectioned into 200 μm slices using a VF-310Z vibratome (Precisionary Instruments, Winchester, MA, USA). Lung slices were maintained in DMEM with 2% antibiotic-antimycotic at 37°C in a humidified atmosphere containing 5% CO_2_. After 24 h of incubation, baseline images were acquired using an inverted phase contrast microscope (Accu-Scope EXI-410). Lung slices were subsequently stimulated with increasing doses of MCh (1 nM to 1 mM) at 3-min intervals, and airway lumen area changes were quantified with ImageJ as a percentage of baseline area. Multiple slices obtained from each mouse were averaged prior to statistical analysis, with each mouse treated as a single biological replicate.

#### Laser-capture microdissection (LCM) assisted ASM isolation and RT-PCR

LCM-assisted ASM isolation was performed as previously described.^27^ Mouse lung tissues were fixed in Carnoy’s solution, paraffin-embedded, sectioned (10 μm), and deparaffinized. ASM regions (0.3 to 1.0 mm^2^) were microdissected using a Zeiss PALM Microbeam LCM system and collected into RNA extraction buffer. RNA was isolated using the PicoPure RNA Isolation Kit (Thermo Scientific), followed by cDNA synthesis (Applied Biological Materials, Canada). qPCR reactions were performed using BlasTaq 2X MasterMix on a QuantStudio 3 system (Thermo Scientific). To verify the specificity of ASM isolation, enrichment of smooth muscle markers (*Acta2* and *Myh11*) and depletion of epithelial (*Cdh1*) and leukocyte (*Ptprc*) markers were assessed (Figure S1).

#### Immunofluorescence

Immunofluorescence was conducted as previously reported.^27^^,^^51^ Lung sections (6 μm) were baked at 56°C for 2 h, followed by deparaffinization, rehydration, and antigen retrieval using citrate buffer (pH 6.0). Sections were then permeabilized with 0.1% Triton X-100 and blocked with 10% goat serum before being incubated overnight at 4°C with α-SMA (1:100) and/or AhR (1:100) primary antibodies. After PBS-T washes, Alexa Fluor-conjugated secondary antibodies (AF-555 for AhR; AF-488 for α-SMA, 1:200) were applied for 2 h at room temperature. Nuclei were counterstained with DAPI, and images were captured using a Zeiss LSM900 Airyscan2 confocal microscope (Oberkochen, Germany). Constant conditions were maintained while capturing all the fluorescence images. To quantify AhR localization in ASM, regions of interest (ROIs) were manually selected by circular selection within ASM layer (three ROIs per field) using Zeiss Zen 3.0 software. Mean fluorescence intensity (MFI) values for each ROI were recorded and normalized to the respective area (MFI/μm^2^).

#### Western blotting of lung tissue homogenates

Lung tissue was homogenized in lysis buffer containing protease inhibitors. Protein concentration was determined, and 30 μg per sample was loaded for SDS-PAGE. Western blotting was performed as described previously.^27^^,^^51^^,^^52^ Blots were probed for AhR, α-SMA, vimentin, collagen-I, β-actin, and GAPDH, followed by IRDye secondary antibodies and detection using a LI-COR NIR imaging system. Densitometric analysis was performed with Image Studio v6.1. Protein band intensities were normalized to β-actin or GAPDH and expressed as relative fold-change versus vehicle. Membranes were reprobed for multiple targets.

#### Real-time quantitative polymerase chain reaction (RT-qPCR)

Total RNA from lung tissue was extracted using the Quick-RNA MiniPrep Kit (Zymo Research, Irvine, CA, USA) and quantified via Synergy HTX Multi-Mode Plate Reader (BioTek, VT, USA). 1 μg RNA was reverse-transcribed using the OneScript Plus cDNA Synthesis Kit (Applied Biological Materials, Canada). qPCR reactions were performed using BlasTaq 2X MasterMix on a QuantStudio 3 system (Thermo Scientific). Relative gene expression was normalized to S16 using the ΔΔCt method, with PBS-treated controls as the reference (fold change = 1). The primers used are listed in key resources table.

#### RNA-sequencing

Approximately 25 mg of frozen lung tissue was homogenized in Tri Reagent (Cat# TR 118, Molecular Research Center, Cincinnati, OH). Total RNA was treated with DNase and isolated using the Qiagen RNeasy Mini Kit (Cat# 74106, Qiagen, Germantown, MD), followed by RNA cleaned up and concentration with the Zymo RNA Clean and Concentrator kit (Cat# R1013, Zymo Research, Irvine, CA). RNA concentration was determined using a Nanodrop spectrophotometer and RNA integrity was assessed with the Agilent 2200 TapeStation system (Agilent Technologies, Santa Clara, CA). All samples exhibited RNA integrity numbers of 8 or greater, indicating high quality RNA with minimal degradation. For cDNA library preparation, 1 μg of total RNA per sample was amplified using the Illumina Stranded mRNA Prep kit (Cat# 20040534 (Stranded mRNA Prep, Ligation) and 20091655 (indexes) and protocol no. 100 000 012 4518 v03, Illumina, San Diego, CA). Libraries were cleaned using the Zymo DNA Clean Up and Concentrator kit (Cat# D4014, Zymo) and purified with Ampure XP beads (Cat# A63881, Beckman Coulter Life Sciences, Indianapolis, IN). Library quality was evaluated using the Agilent 2200 TapeStation, and quantification was performed with the Collibri Library Quantification Kit (Cat# A38524500; Thermo Fisher Scientific, Carlsbad, CA) on a QuantStudio 6 Flex System (Thermo Fisher Scientific). Quantified sequencing libraries were pooled, loaded onto an Illumina NovaSeq 6000 SP flow cell, and then sequenced using 150 bp paired-end reads plus a single index read.

#### Quality control and read alignment

Data was processed on the Franklin Cluster using custom Python^53^ scripts. Fastq files were checked for quality with FastQC^54^ before and after trimming with bbduk,^55^ and aligned to the GRCm38 Genome^56^ obtained from Gencode^57^ with Hisat2^58^ (--very-sensitive, K=2). Additional QC metrics, file conversion, sorting, indexing, and removal of non-mRNA genes was performed with RSeQC,^59^ and samtools.^60^ Features were counted using HTSeq2^61^ (-t CDS) using annotations also acquired from Gencode.

#### Differential expression analysis

Differential expression analysis was performed in RStudio (v4.5.1) using DESeq2 (v1.50.2), a Bioconductor package, along with other Bioconductor- and CRAN-based libraries.^62^^,^^63^ Raw count matrices were imported and genes with fewer than 10 read counts in at least three samples were filtered prior to analysis to reduce noise from lowly expressed transcripts. DESeq2’s median-of-ratios normalization was applied to account for differences in sequencing depth and library composition across samples.^64^ Variance-stabilizing transformation (VST) was applied for downstream data visualization and quality control analyses.^62^ Differential expression was assessed using the Wald test implemented in DESeq2. Three biologically relevant pairwise comparisons were performed: MA vs PBS to evaluate the effect of mixed allergen exposure, TCDD vs PBS to assess the effect of AhR activation alone, and MA+TCDD vs MA to determine the impact of AhR activation in the context of allergen-induced asthma. Genes were considered differentially expressed with log2 fold change ≥ 1 (≥2-fold change) and a raw *p*-value < 0.05. Multiple-testing correction was not applied at this stage to preserve sensitivity for downstream pathway and network analyses, consistent with recommended practices for exploratory systems-level analyses.^65^

#### Data visualization and quality assessment

All visualization and data assessment were performed using various R packages, including tidyverse, ggplot2, dplyr, pheatmap, ggrepel, RColorBrewer, and biomaRt.^66^^,^^67^^,^^68^^,^^69^^,^^70^^,^^71^^,^^72^ Sample-level quality and reproducibility were determined using principal component analysis (PCA) based on variance-stabilized expression values (Figure S7). Volcano plots were generated to visualize differentially expressed genes (DEGs), with significantly regulated genes (|log2FC| ≥1, *p* <0.05) highlighted. Heatmaps displaying the top 50 most significantly DEGs for each comparison were generated using hierarchical clustering based on Pearson correlation distance, with row-wise Z-score normalization applied to emphasize relative expression patterns across samples.

#### Pathway and network analysis

DEG lists from each comparison were independently analyzed using QIAGEN Ingenuity Pathway Analysis (IPA; QIAGEN Inc.) to identify enriched canonical pathways, upstream regulators, and molecular interaction networks.^73^ Canonical pathway analysis was restricted to Reactome-curated pathways to reduce redundancy and ensure biologically well-defined signaling annotations.^74^ IPA enrichment analyses were performed using right-tailed Fisher’s exact test, and pathway activation states were predicted using the activation z-score, which indicates directional regulation based on relation between gene expression changes and available biological relationships within the IPA Knowledge Base.^73^ Gene interaction networks were generated based on experimentally validated and curated molecular interactions. Networks were prioritized according to network score and biological relevance to airway inflammation, immune regulation, and tissue remodeling. Upstream and downstream regulatory networks were visualized to capture regulatory drivers and transcriptional consequences associated with MA-induced asthma and AhR activation.

### Quantification and statistical analysis

Each experimental group included a minimum of six biological replicates. Results are reported as mean ± SEM. Statistical analyses were conducted using GraphPad Prism v10.5.0 (GraphPad Software, San Diego, CA, USA). Differences between groups were analyzed using either an unpaired t-test or one/two-way ANOVA followed by Tukey’s post hoc test for multiple comparisons. A *p* value < 0.05 was considered statistically significant and indicated as ∗*p* < 0.05, ∗∗*p* < 0.01, and ∗∗∗*p* < 0.001. Statistical methods for RNA-sequencing data are described separately in the differential expression analysis section.
