# Supplementary material for: Pharmacological AhR activation attenuates airway hyperresponsiveness and remodeling in a mixed allergen asthma model
Source: iScience. 2026 Oct 17;29(11):117842. doi: 10.1016/j.isci.2026.117842 (PMC13696118; doi:10.1016/j.isci.2026.117842)
Supplement: Supplementary file 1 [file mmc1.pdf]

iScience, Volume *29*

Supplemental information

Pharmacological AhR activation attenuates airway hyperresponsiveness and remodeling in a mixed allergen asthma model

Mohammad Irshad Reza, Premanand Balraj, Ashish Kumar, Kathryn M. Heyob, Joshua Walum, Rodney D. Britt Jr., and Venkatachalem Sathish

Supplemental information

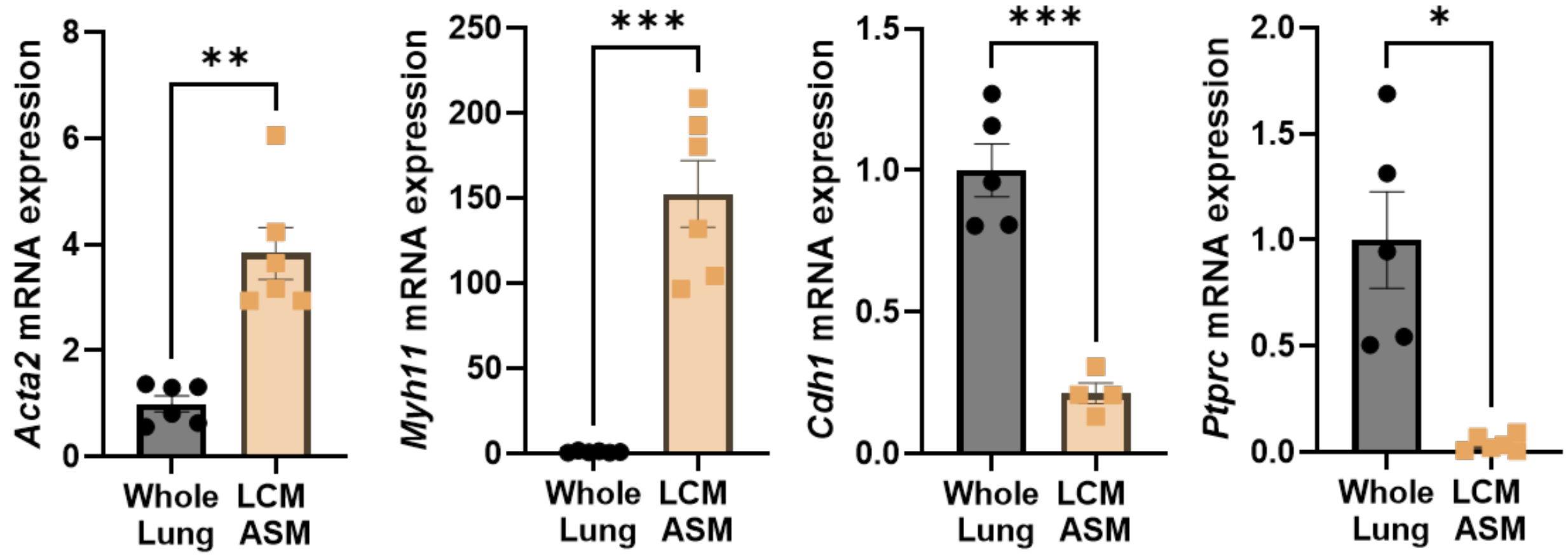

Figure S1. **LCM ASM enrichment validation, Related to Figure 5**. RT-qPCR analysis confirming enrichment of ASM (high *Acta2* and *Myh11* expression) with minimal epithelial (*Cdh1*) and leukocyte (*Ptprc*) contamination in the laser capture microdissected ASM layer compared with whole lung tissue. Data were evaluated using unpaired Student's t-test. Mean ± SEM (n=4-6 mice/group); *P < 0.05, **P < 0.01, ***P < 0.001.

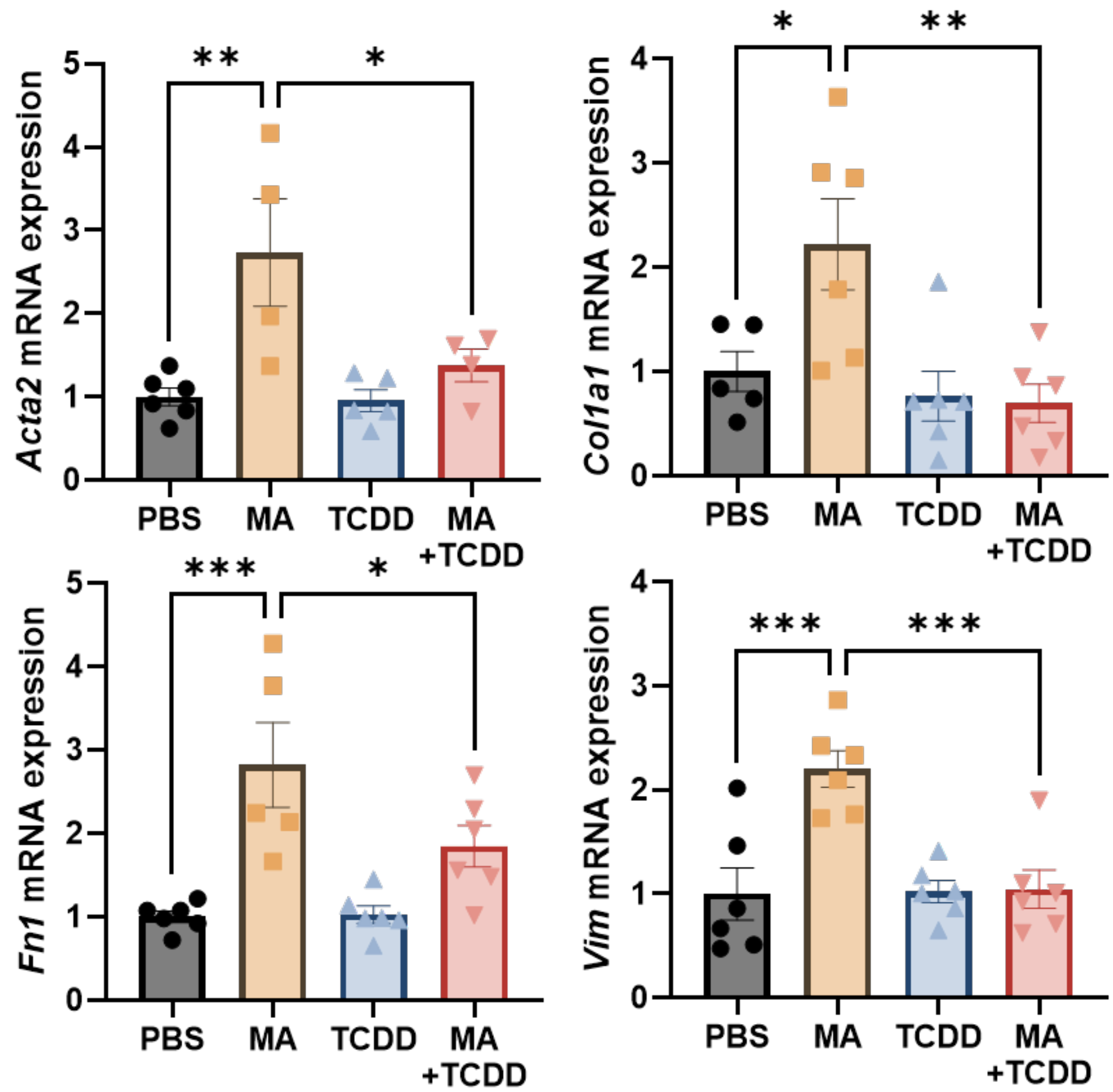

Figure S2. **Remodeling associated gene expression in LCM isolated ASM, Related to Figure 3**. RT-qPCR analysis of remodeling-associated genes in laser capture microdissected ASM. Data were analyzed using one-way ANOVA followed by Tukey's post hoc test. Mean ± SEM (n=4-6 mice/group); *P < 0.05, **P < 0.01, ***P < 0.001.

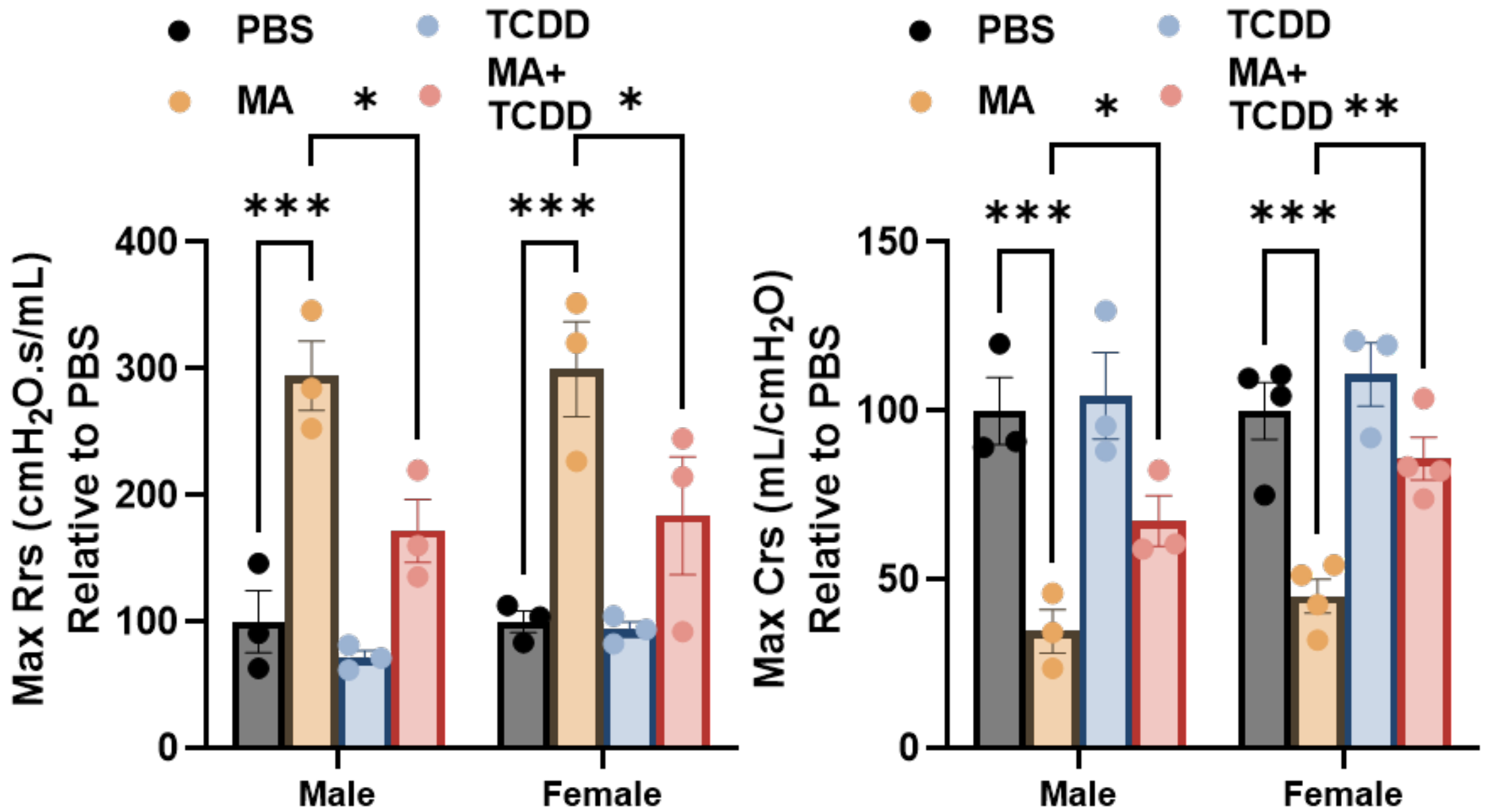

Figure S3. **Sex stratified lung function analysis, Related to Figure 1**. Maximum airway resistance (Rrs) and compliance (Crs) following methacholine challenge are shown separately for male and female mice. TCDD-mediated AhR activation reduced airway resistance and improved lung compliance in both sexes. Data were analyzed using two-way ANOVA followed by Tukey's post hoc test. Mean ± SEM (n=3-4 mice/sex/group); *P < 0.05, **P < 0.01, ***P < 0.001.

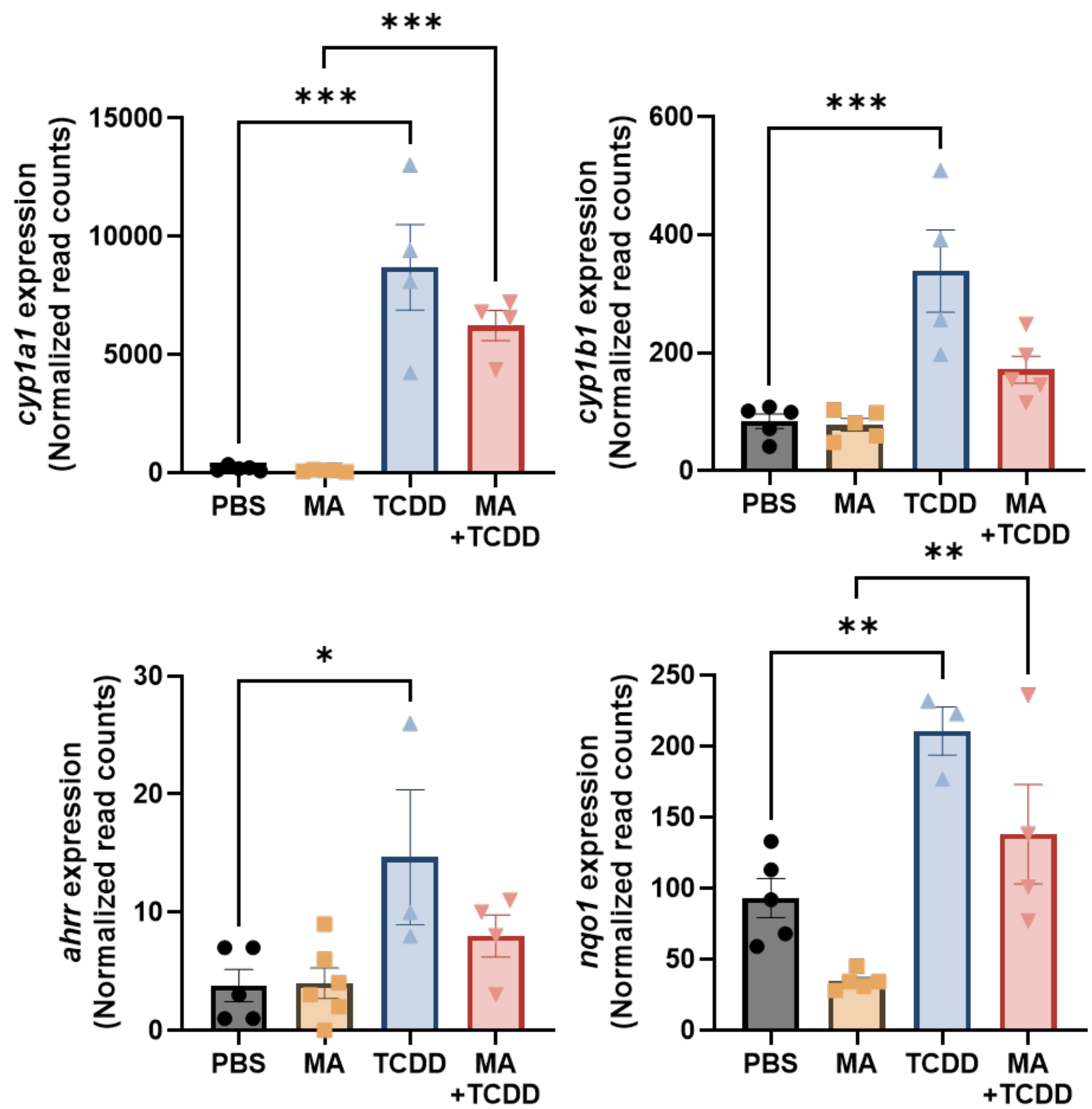

Figure S4. **Canonical AhR target gene expression, Related to Figures 5 and 6**. Canonical AhR target gene expression following mixed allergen exposure and AhR activation. RNA-seq normalized expression of *Cyp1a1, Cyp1b1, Ahrr*, and *Nqo1* across PBS, MA, TCDD, and MA+TCDD groups. MA exposure alone produced little reduction of canonical AhR target genes, whereas TCDD treatment markedly increased *Cyp1a1, Cyp1b1, Ahrr*, and *Nqo1* expression, confirming effective AhR activation in vivo. Data represent normalized RNA-seq read counts. Statistical analysis was performed using one-way ANOVA followed by Tukey's multiple comparisons test. Mean ± SEM (n=4-6mice/group); *P < 0.05, **P < 0.01, ***P < 0.001.

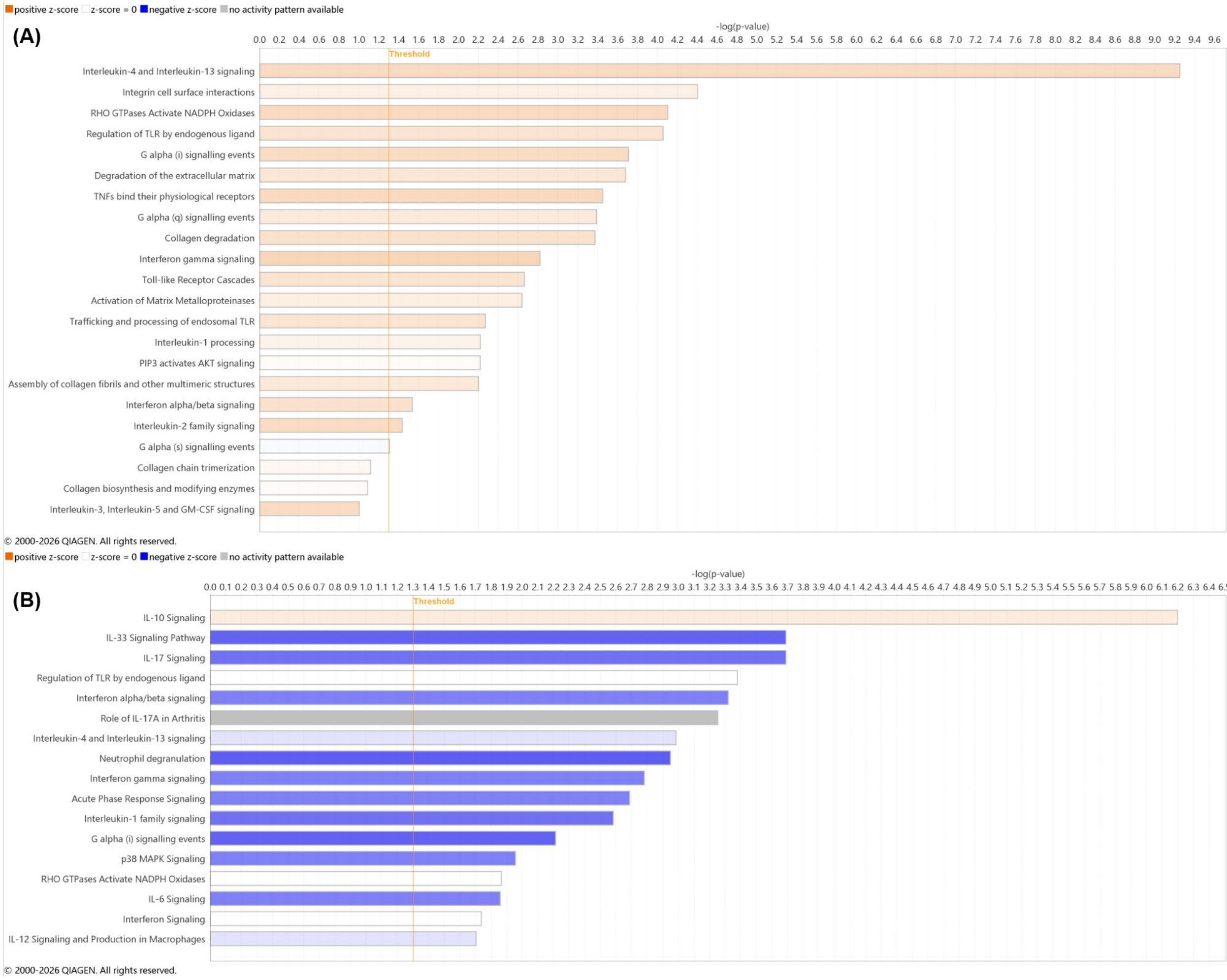

Figure S5. FDR sensitivity analysis of canonical pathways, Related to Figure 7. Sensitivity analysis of canonical pathways identified using FDR-adjusted differentially expressed genes. Ingenuity Pathway Analysis (IPA) of FDR-adjusted differentially expressed genes demonstrating (A) enrichment of inflammatory and airway remodeling pathways in the MA vs PBS comparison and (B) suppression of inflammatory, innate immune, and remodeling pathways in the MA+TCDD vs MA comparison following AhR activation. Pathway significance is represented as -log(p-value) and predicted pathway activity is indicated by the activation z-score, where positive values indicate predicted activation and negative values indicate predicted inhibition. n = 5-6 mice per group.

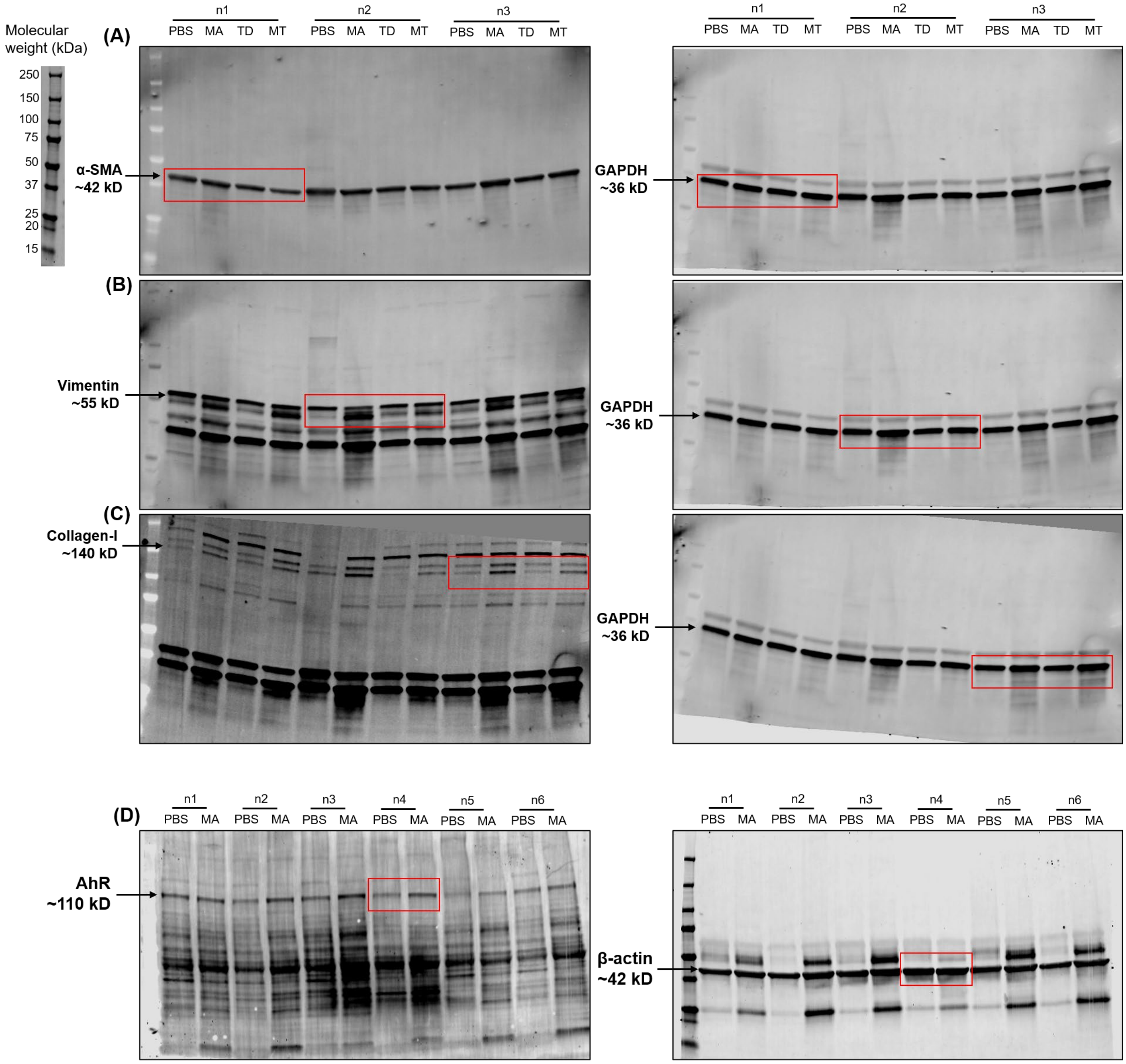

Figure S6. **Unprocessed western blot images, Related to Figures 3 and 5**. Raw blots of (A) α-SMA and corresponding GAPDH, (B) Vimentin and corresponding GAPDH, (C) Collagen-I and corresponding GAPDH, (D) AhR and corresponding β-actin. TD: TCDD; MT: MA+TCDD, n = biological replicate.

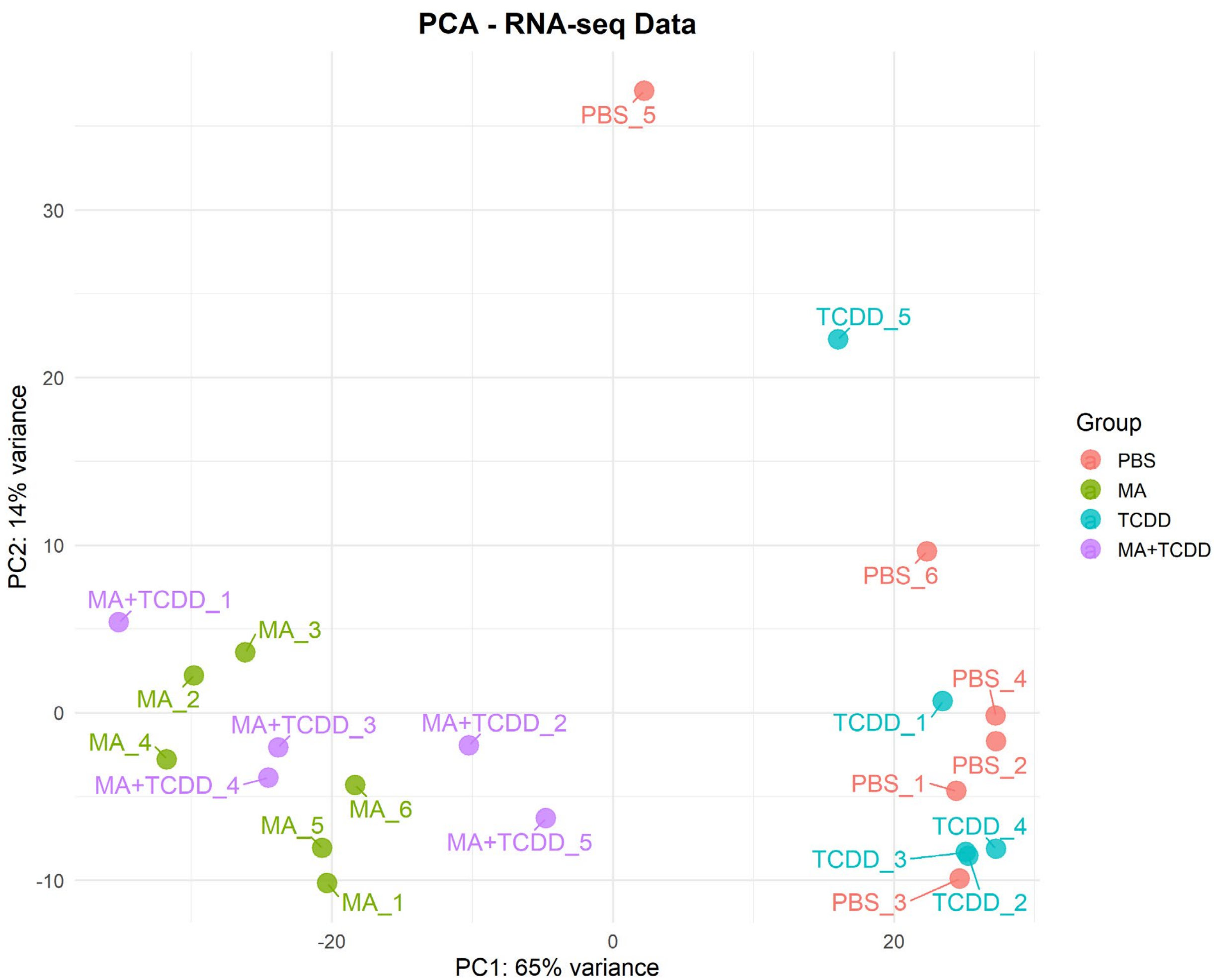

Figure S7. Principal component analysis of lung RNA sequencing samples, Related to Figure 6. PCA was performed using normalized gene expression data from lung tissues of PBS, MA, TCDD, and MA+TCDD-treated mice. PC1 and PC2 account for 65% and 14% of the total variance, respectively. Samples cluster by experimental group, indicating high within-group consistency. Separation along PC1 reflects the dominant transcriptional impact of mixed allergen–induced asthma, while variation along PC2 reflects treatment-associated modulation of lung gene expression.

Table S1. Canonical pathways and associated differentially expressed genes identified by Ingenuity Pathway Analysis in MA-induced asthmatic mice, Related to Figure 7A.

Canonical Pathways	-log(p-value)	z-score	Molecules
Interleukin-4 and Interleukin-13 signaling	8.26	2.828	BATF, CCL2, CCL22, CDKN1A, F13A1, FCER2, FGF2, FSCN1, HMOX1, IGHG1, IL10, IL18, IL1A, IL1B, IL4, IL6, IRF4, ITGAM, ITGAX, ITGB2, LCN2, MMP9, NOS2, OSM, PIM1, SOCS1, SOCS3, TGFB1, TNF, TNFRSF1B, TWIST1, VEGFA
G alpha (i) signaling events	5.17	2.795	ADCY5, ADCY8, ADORA3, ADRA2A, AGT, BDKRB1, C3, C3AR1, CCL20, CCL5, CCR1, CCR10, CCR2, CCR3, CCR4, CCR6, CHRM2, CXCL16, CXCL9, CXCR2, CXCR6, GAL, GRM6, HCAR1, HCAR2, NPY, OPN3, P2RY14, PF4, PSAP, PTGER3, RGS1, RGS10, RGS11, RGS13, RGS16, RGS7
RHO GTPases Activate NADPH Oxidases	4.21	3.162	CYBA, NCF1, NCF2, NCF4, NOXA1, NOXO1, PRKCD, RAC2, S100A8, S100A9
Regulation of TLR by endogenous ligand	3.98	2.333	APOB, CD14, FGG, GSDME, S100A8, S100A9, SFTPD, TLR1, TLR6
Xenobiotic Metabolism AHR Signaling Pathway	3.92	0.577	Aldh1a1, ALDH1A3, ALDH1B1, ALDH1L2, ALDH5A1, ALDH6A1, Gsta1 (includes others), Gsta3, Gstm1, GSTZ1, IL1A, IL1B, IL6, NFKB2, NQO1, PON1, RELB, TNF
G alpha (q) signaling events	3.91	2.268	ADRA1A, ADRA1D, AGT, AVPR1A, BDKRB1, EDN3, EDNRB, F2RL2, FFAR2, GNA15, GPR65, GPR68, GPRC6A, HRH1, HTR2B, LTB4R, LTB4R2, NTS, P2RY1, P2RY2, P2RY6, PLCB2, PTAFR, PTGFR, RGS1, RGS13, RGS16, XCR1
Collagen degradation	3.83	2.5	COL12A1, COL17A1, COL3A1, COL5A2, COL6A5, COL6A6, CTSB, CTSD, CTSK, CTSL, MMP12, MMP13, MMP14, MMP19, MMP8, MMP9
Degradation of the extracellular matrix	3.65	2.357	ADAM8, ADAMTS4, CAPN13, CAPN9, CD44, CTSK, CTSL, CTSS, DCN, FBN2, HTRA1, MMP12, MMP13, MMP14, MMP19, MMP8, MMP9, SPP1
Integrin cell surface interactions	3.62	1.789	CD44, COL3A1, COL5A2, COL6A5, COL6A6, FGG, ICAM5, ITGA7, ITGA8, ITGAE, ITGAM, ITGAX, ITGB2, ITGB8, KDR, LUM, SPP1, THBS1, TNC, VTN

TNFs bind their physiological receptors	3.52	3.162	LTA, TNFRSF11B, TNFRSF18, TNFRSF1B, TNFRSF4, TNFRSF8, TNFRSF9, TNFSF11, TNFSF14, TNFSF8
Interferon gamma signaling	2.72	3.5	CD44, CIITA, FCGR1A, HLA-A, HLA-DQA1, IFNG, IFNGR2, IRF4, IRF5, IRF7, OAS3, PRKCD, PTAFR, SOCS1, SOCS3, TRIM29
Toll-like Receptor Cascades	2.71	2.333	CD14, ITGAM, ITGB2, LY86, SFTPD, TICAM2, TLR1, TLR5, TLR6
PIP3 activates AKT signaling	2.29	0.392	BTC, CD28, CD80, CD86, CDKN1A, ESR2, FGF1, FGF2, FGF23, FGFR3, FGFR4, FLT3, FOXO6, ICOS, IL1RL1, IL33, IRS1, KITLG, KLB, LCK, NRG2, NTRK2, PIK3CG, PIK3R5, RAC2, VAV1
Trafficking and processing of endosomal TLR	2.23	2.236	CTSB, CTSK, CTSL, CTSS, LGMN
Interleukin-1 processing	2.18	1	IL18, IL1A, IL1B, NFKB2
Activation of Matrix Metalloproteinases	2.18	1.89	CTSK, MMP13, MMP14, MMP16, MMP25, MMP8, MMP9
Assembly of collagen fibrils and other multimeric structures	2.03	1.732	COL12A1, COL17A1, COL3A1, COL5A2, COL6A5, COL6A6, CTSB, CTSL, CTSS, LOX, MMP13, MMP9
Interleukin-2 family signaling	1.56	3	CSF2, CSF2RB, HAVCR2, IL21R, IL2RA, IL2RB, IL5, INPP5J, LCK
Interferon alpha/beta signaling	1.48	2.53	HLA-A, IRF4, IRF5, IRF7, MX1, OAS3, PSMB8, RSAD2, SOCS1, SOCS3

Table S2. Canonical pathways and associated differentially expressed genes identified by Ingenuity Pathway Analysis in TCDD treated MA-induced asthmatic mice, Related to Figure 7B.

**Canonical Pathways**	**-log (p-value)**	**z-score**	**Molecules**
Interleukin-4 and Interleukin-13 signaling	3.53	-0.816	CCL2, HMOX1, IL10, IL1B, MMP9, NOS2
Xenobiotic Metabolism AHR Signaling Pathway	3.49		ALDH1A3, ALDH1L2, CYP1A1, IL1B, NQO1
Interferon gamma signaling	3.4	-1.342	FCGR1A, IFNG, IRF7, OAS3, TRIM17
Regulation of TLR by endogenous ligand	3.12		GSDME, S100A8, S100A9
Interferon alpha/beta signaling	2.98	-2	IRF7, ISG15, OAS3, RSAD2
G alpha (i) signaling events	2.42	-2.449	CCL5, CXCL9, CXCR2, HCAR2, P2RY13, PF4
RHO GTPases Activate NADPH Oxidases	1.73		S100A8, S100A9
Interleukin-1 family signaling	1.61	-2	IL18BP, IL1B, IL1R2, IL1RN
Gene and protein expression by JAK-STAT signaling after IL-12 stimulation	1.39		IFNG, IL10
